# Health inequalities in risk of SARS-CoV-2 infection in England’s second wave: population-based cross-sectional analysis from the REACT-1 study

**DOI:** 10.1136/bmjph-2025-002596

**Published:** 2026-01-05

**Authors:** Haowei Wang, Kylie E. C. Ainslie, Caroline E. Walters, Oliver Eales, David Haw, Christina Atchison, Claudio Fronterre, Peter J. Diggle, Deborah Ashby, Graham Cooke, Wendy Barclay, Helen Ward, Ara Darzi, Christl A. Donnelly, Steven Riley, Paul Elliott

**Affiliations:** 1 School of Public Health, Imperial College London, London, UK; 2 MRC Centre for Global Infectious Disease Analysis and Abdul Latif Jameel Institute for Disease and Emergency Analytics, Imperial College London, London, UK; 3 Centre for Infectious Disease Control, National Institute for Public Health and the Environment, Bilthoven, Netherlands; 4 Bennett Institute for Applied Data Science, University of Oxford, Oxford, UK; 5 Department of Mathematical Sciences/Institution of Infection, Veterinary and Ecological Sciences, University of Liverpool, Liverpool, UK; 6 CHICAS, Lancaster Medical School, Lancaster University, Lancaster, UK; 7 Department of Infectious Disease, Imperial College London, London, UK; 8 Imperial College Healthcare NHS Trust, London, UK; 9 National Institute for Health Research Imperial Biomedical Research Centre, London, UK; 10 Imperial College London Institute of Global Health Innovation, London, UK; 11 Department of Statistics, University of Oxford, Oxford, UK; 12 MRC Centre for Environment and Health, School of Public Health, Imperial College London, London, UK; 13 Health Data Research (HDR) UK, Imperial College London, London, UK; 14 UK Dementia Research Institute, Imperial College London, London, UK

**Keywords:** Epidemiology, SARS-CoV-2, Public Health, COVID-19

## Abstract

**Objectives:**

The rapid spread of SARS-CoV-2 infection caused high levels of hospitalisation and deaths in late 2020 and early 2021 during the second wave in England. COVID-19 disease during this period was associated with marked health inequalities across ethnic and sociodemographic subgroups. In this paper, we aim to investigate and quantify inequalities in the risk of SARS-CoV-2 infection across ethnic and sociodemographic subgroups during a key period before widespread vaccination, thereby identifying the populations that bore a disproportionate burden of risk.

**Methods:**

We analysed risk factors for test-positivity for SARS-CoV-2, based on self-administered throat and nose swabs in the community during rounds 5–10 of the REal-time Assessment of Community Transmission-1 (REACT-1) study between 18 September 2020 and 30 March 2021.

**Results:**

Compared with the White ethnicity, people of Asian and the Black ethnicity had a higher risk of infection during rounds 5–10, with odds of 1.45 (1.27, 1.66) and 1.34 (1.11, 1.63), respectively, adjusted for demographic factors including age, sex, region, key work status, ethnicity and deprivation. Among ethnic subgroups, the highest and the second-highest odds were found in Bangladeshi and Pakistani participants at 3.28 (2.24, 4.80) and 2.12 (1.70, 2.64), respectively, when compared with the White British participants. People in larger (compared with smaller) households had higher odds of infection. Healthcare workers with direct patient contact and care home workers showed higher odds of infection compared with other essential/key workers. Additionally, the odds of infection among participants in public-facing activities or settings were greater than among those not working in those activities or settings.

**Conclusion:**

Our findings highlight the differences in the risk of SARS-CoV-2 infection in a global north population during a period when the risk of infection was high, and there were substantial levels of social mixing. Planning for future waves of severe respiratory pathogens should include policies to reduce inequality in the risk of infection by ethnicity, household size and occupational activity in order to reduce inequality in disease.

**WHAT IS ALREADY KNOWN ON THIS TOPIC**

- Extensive studies have described the relationship between socio-demographic factors and SARS-CoV-2 outcomes such as cases, hospitalisations and deaths, rather than SARS-CoV-2 infection.
- Far less evidence is available from community-based studies based on SARS-CoV-2 infection status rather than symptoms, with the time frame of studies which do contain this evidence mainly focused on the period of the first wave of infection, the beginning of the second wave or the rollout of the first dose of the vaccine after the second wave period.
- We did not find studies that covered the critical period of the second wave in England when levels of social mixing were high, but no vaccine was available.

**WHAT THIS STUDY ADDS**

- We show health inequalities across ethnic and sociodemographic subgroups during a key period: before widespread vaccination, but, largely, not during the period of stringent social distancing.
- We observed substantial ethnic and occupational differences in the risk of SARS-CoV-2 infection.
- Minority ethnic groups, including those of Bangladeshi and Pakistani ethnicity, had an excess risk of infection compared with the White British population.
- Healthcare workers, care home workers and people who work in public-facing activities or settings were associated with higher odds of infection.
- The risk of SARS-CoV-2 infection increased monotonically as household size increased, and more deprived neighbourhood areas were associated with a higher risk of infection.

**HOW THIS STUDY MIGHT AFFECT RESEARCH, PRACTICE OR POLICY**

- Our findings highlight the differences in the risk of SARS-CoV-2 infection in a global-north population during a period when the risk of infection was high, and there were substantial levels of social mixing.
- Planning for future waves of severe respiratory infection should explicitly aim to reduce inequalities in infection in order to reduce inequality in disease.

## Introduction

 The COVID-19 pandemic caused a substantial increase in hospitalisations and mortality after its emergence in China in late December 2019 and its subsequent spread.^1^ Populations continued to experience multiple waves of infection, with substantial reactive changes in social distancing still occurring in some countries either spontaneously or as a result of explicit government regulations for the following 2–3 years.^2,3^ These social distancing measures exacerbated pre-existing structural inequalities, disproportionately impacting disadvantaged groups. The uneven consequences observed across different sociodemographic groups have been widely attributed to interactions between demographic characteristics, such as sex, age, ethnicity and geographic region, and underlying systemic disparities, including unequal access to healthcare, employment insecurity and crowded living conditions.^4,5^ For example, lower-income individuals often have jobs that cannot be done remotely, increasing their risk of exposure to infection and subsequent economic hardship. Similarly, overcrowded housing conditions in economically disadvantaged areas made it difficult to maintain physical distancing, increasing the risk of virus transmission. Investigating these structural inequalities and their interactions with demographic factors, as they relate to infection status, is essential for policymakers to effectively mitigate health disparities during public health emergencies.

In order to enable isolation and quarantine policies, routine testing programmes were implemented nationwide, which mainly targeted individuals who reported symptoms.^6^ While targeted testing provides valuable surveillance, sub-clinical cases that are likely to contribute substantially to transmission are not captured. Furthermore, the uptake of testing may be limited by test capability and geographic, demographic and social factors, and thus not be fully representative even among those eligible for tests. Therefore, tracking the characteristics and underlying inequalities of individuals at risk of SARS-CoV-2 infections through a proactive survey rather than through passive testing can provide additional evidence for policymaking.^7^ In the UK, large representative population-based studies were conducted to understand better the underlying trend of the epidemic and risk factors.^8–11^

There is considerable evidence across many geographies that minority ethnic groups were at increased per-person risk of severe disease and death from COVID-19. However, there remains considerable uncertainty as to what extent this was driven by differential exposure,^12–14^ susceptibility,^15–17^ infection,^15,18,19^ access to testing^20,21^ or differential access to healthcare.^21–23^ Reducing disease disparities during future similar emergencies will only be possible if we understand why they arose during this pandemic.

The REal time Assessment of Community Transmission-1 (REACT-1) study was designed to be representative of the population of England as a whole.^24^ The REACT-1 study tracked the spread of the virus through a total of 19 cross-sectional surveys to collect self-administered throat and nose swabs which were tested for the presence of SARS-CoV-2 by reverse transcriptase polymerase chain reaction.^24^ Participants were asked to take part in the study regardless of whether they were symptomatic or not. The first round of the study began on 1 May 2020, and each new round thereafter was conducted at approximately monthly intervals, with a 2-week break between the end of the previous round and the beginning of the next round. In each round up to round 12, participants were sampled randomly with a similar achieved sample size from 315 lower-tier local authorities (LTLAs).

Here, we present findings on risk factors and quantify the differences in levels of SARS-CoV-2 infection for rounds 5–10 between mid-September 2020 and the end of March 2021, which covered the period of the second wave in England. Results on risk factors from the previous four rounds between May and the beginning of September 2020 have been published previously.^25^

## Methods

### Data collection

The detailed study protocol of the REACT-1 study has been reported elsewhere.^24^ In brief, we randomly selected individuals aged 5 years and above from the National Health Service (NHS) patient list to send personalised invitations, balanced across 315 LTLAs. A key feature of the sampling design is that only one person was selected per household for each round, which ensures the independence of observations with respect to household membership. Participants received test kits and instructions delivered by post and conducted self-administered throat and nose swabs (parent/guardian administered at ages 5–12 years). In addition to the information registered on the NHS list (name, age, sex and address), participants were requested to complete an online or telephone questionnaire to provide other detailed information, for example, employment type, ethnicity, household size, whether they were in contact with a confirmed or suspected COVID-19 case and symptom status.

### Socio-demographic groups

Study questionnaires are available on the REACT 1 Study Materials webpage on the Imperial College London website.^26^

In order to investigate prevalence by subgroups, we converted continuous variables such as age into categories and regrouped some categorical variables into fewer categories by combining similar categories. We divided age into eight age groups as follows: 5–12, 13–17, 18–24, 25–34, 35–44, 45–54, 55–64 years and 65 years or older. 19 ethnic groups were re-grouped into five broad categories: White, Asian, Black, Mixed ethnic group and Other ethnic groups for some analyses. Participants were asked to report the number of children or young people between the ages of 0 and 17 and adults aged over 18 years who currently live in or spend part of the week in the household of the respondent. We obtained the total household size by adding these numbers. We then created a new categorical variable for household size with six categories (one person, two people, three people, four people, five people and six or more people), based on the number of people in each household. For the variable on COVID-19 case contact, participants answered whether they were in contact with a confirmed/tested or suspected COVID-19 case. We did not further re-categorise this variable.

We asked participants if they worked as healthcare workers or care home workers or other key workers. If they did not work as a key worker, they were asked if they were engaged in one or more public-facing activities or settings (11 different activities for rounds 5–8, 12 activities for rounds 9 and 10). In addition, participants were asked about their employment status (employed in full-time jobs, employed in part-time jobs, self-employed, unemployed, retired from work). We combined employment and key worker answers into a new single variable which we called key worker status that could take one of the following four values: (1) healthcare or care home worker, (2) other essential/key workers, (3) other workers and (4) not full-time, part-time or self-employed.

We also regrouped symptom status during the month before the test. Participants were classified as ‘Classic COVID-19 symptoms’ if they had experienced one or more of the following symptoms in the past month: loss or change of a sense of smell or taste, persistent cough or fever. Other symptomatic people who did not show classic COVID-19 symptoms in the past month were classified as 'Other symptoms’. Participants who did not feel unwell and did not present any of the listed symptoms were classified as 'No symptoms’. Diarrhoea and other gastrointestinal symptoms, although increasingly recognised as part of the broader COVID-19 clinical spectrum,^27,28^ were not included in the 'Classic symptoms’ group and were instead classified under 'Other symptoms’ in accordance with the UK public health case definition used during the study period.

### Statistical analysis

We estimated the unweighted prevalence as the proportion of the total samples that were positive. To correct for non-response bias and unequal selection probabilities that occurred in the sampling process, we used Random Iterative Method (RIM) weighting^29^ to adjust unweighted prevalence with pre-calculated survey weights and obtain prevalence estimates representative of the population in England. The method and the process of applying it to REACT-1 data have been described elsewhere.^25^ RIM weights were based on age, sex, LTLA response, ethnic group and Index of Multiple Deprivation^30^ deciles.

We fitted multivariable logistic regression models to investigate whether particular subgroups were more likely to test positive for SARS-CoV-2. The binary outcome variable was swab-positivity (yes or no), while age, sex, region, key worker status, broad categories of ethnicity, household size and deprivation were included as explanatory variables. The OR was reported along with corresponding 95% CIs. We conducted two types of multivariable logistic regression analyses: (1) independently associated analysis and (2) mutually adjusted analysis. In the first analysis, each socio-demographic variable (eg, key worker status, broad categories of ethnicity, household size and deprivation) was adjusted for age and sex only. This allowed us to determine the independent association of each socio-demographic variable with swab-positivity, controlling solely for age and sex. In the mutually adjusted analysis, all sociodemographic variables were included in one model, mutually adjusted for age, sex and each other. This provided a comprehensive assessment of the combined effects of all included variables on the likelihood of testing positive for SARS-CoV-2.

The associations of swab-positivity with detailed categories of occupation and ethnicity subgroups were also quantified using logistic regression models: for detailed occupation, the model was adjusted for age, sex, region, broad categories of ethnicity and deprivation; and for ethnicity subgroups, the model was adjusted for age, sex, region, broad categories of key worker status and deprivation. The reason for using different explanatory variables for detailed occupation and detailed ethnicity is that broad key worker status and broad ethnicity are derived from detailed occupation and detailed ethnicity, respectively, making them dependent. Including both broad and detailed categories of occupation and ethnicity in the same model would introduce collinearity, leading to results that would be difficult to interpret. Therefore, we used distinct sets of covariates for each model to avoid this issue and ensure accurate estimation of the associations.

To assess the overall risk for subgroups across rounds 5–10, we pooled log-odds ratios from each individual round using the inverse-variance method.^31^

As the rollout of the vaccination programme in England began on 8 December 2020,^32^ we included vaccination-related questions in the questionnaire from round 8 (January 2021) and asked participants for consent to longer-term follow-up through linkage to their NHS records. Here, our primary objective is not to investigate patterns of vaccination and its impact on infection. We conducted a sensitivity analysis of other results using data from the vaccination questions. Due to the low number of people vaccinated in round 8, we only linked to NHS vaccination records during rounds 9 and 10 with a match to 114 747 (69%) participants in round 9 and 98 983 (70%) participants in round 10. We conducted a logistic regression analysis based on the linked data, adjusting for age, sex, region, key worker status, broad categories of employment, broad categories of ethnicity, household size and deprivation. Vaccination status was included as an explanatory variable in the model to determine its impact on swab-positivity rates.

## Results

### Levels of swab-positivity during the second wave in England

England experienced a large second wave of SARS-CoV-2 infection between September 2020 and March 2021, with rising overall swab positivity from September 2020 to January 2021, followed by a decline to March 2021 (figure 1 and online supplemental table S1). In wave two between 18 September 2020 and 30 March 2021, 7052 samples were positive overall from 977 245 swabs.

**Figure 1:**
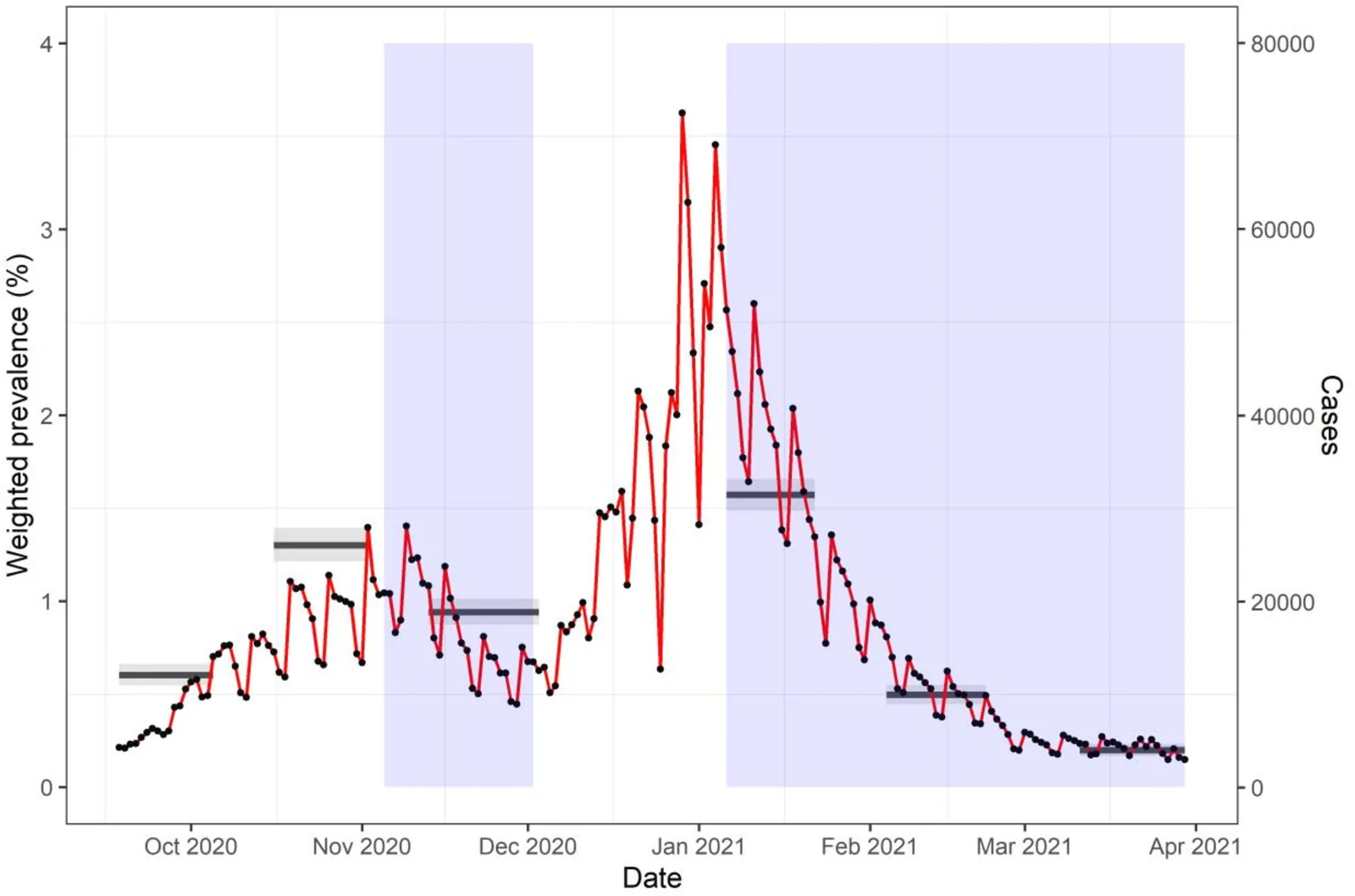
Comparison of overall weighted prevalence as measured by REACT-1 to surveillance case data (Public Health England, PHE). Overall weighted prevalence for rounds 5–10 of the REACT-1 study is in black solid lines (left-hand side y-axis) with a 95% CI (grey shaded boxes). The temporal dynamic of surveillance data is shown by black points and the red solid line (right-hand side y-axis). The right y-axis is scaled by 20 000 per case. Blue-shaded areas indicate the time period of the second and the third lockdowns in England. REACT-1, REal time Assessment of Community Transmission-1.

### Ethnicity

Self-identified broad classes of ethnicity were a substantial risk factor for testing positive during this period of the epidemic in England and remained important even after adjustment for household size and level of local neighbourhood deprivation. Broad classes of ethnicity were defined by participants self-identifying as one of the White, Asian, Black, Mixed and Other ethnic groups. The Asian ethnic group showed the highest weighted prevalence consistently during the second wave, while the White ethnic group was at the lowest level (figure 2A, online supplemental table S2). Similar patterns of higher infection prevalence in the Black and Asian ethnic groups were seen in England in subsequent REACT-1 rounds.^33–41^ Across rounds 5 to 10, the odds of being swab-positive were 1.45 (1.27, 1.66) and 1.34 (1.11, 1.63) times greater for people of Asian and the Black ethnicity respectively, compared with the White ethnicity, whereas the odds were 0.93 (0.77, 1.11) times lower for people of the Mixed ethnic group than the White ethnic group (figure 2A, online supplemental table S3).

**Figure 2:**
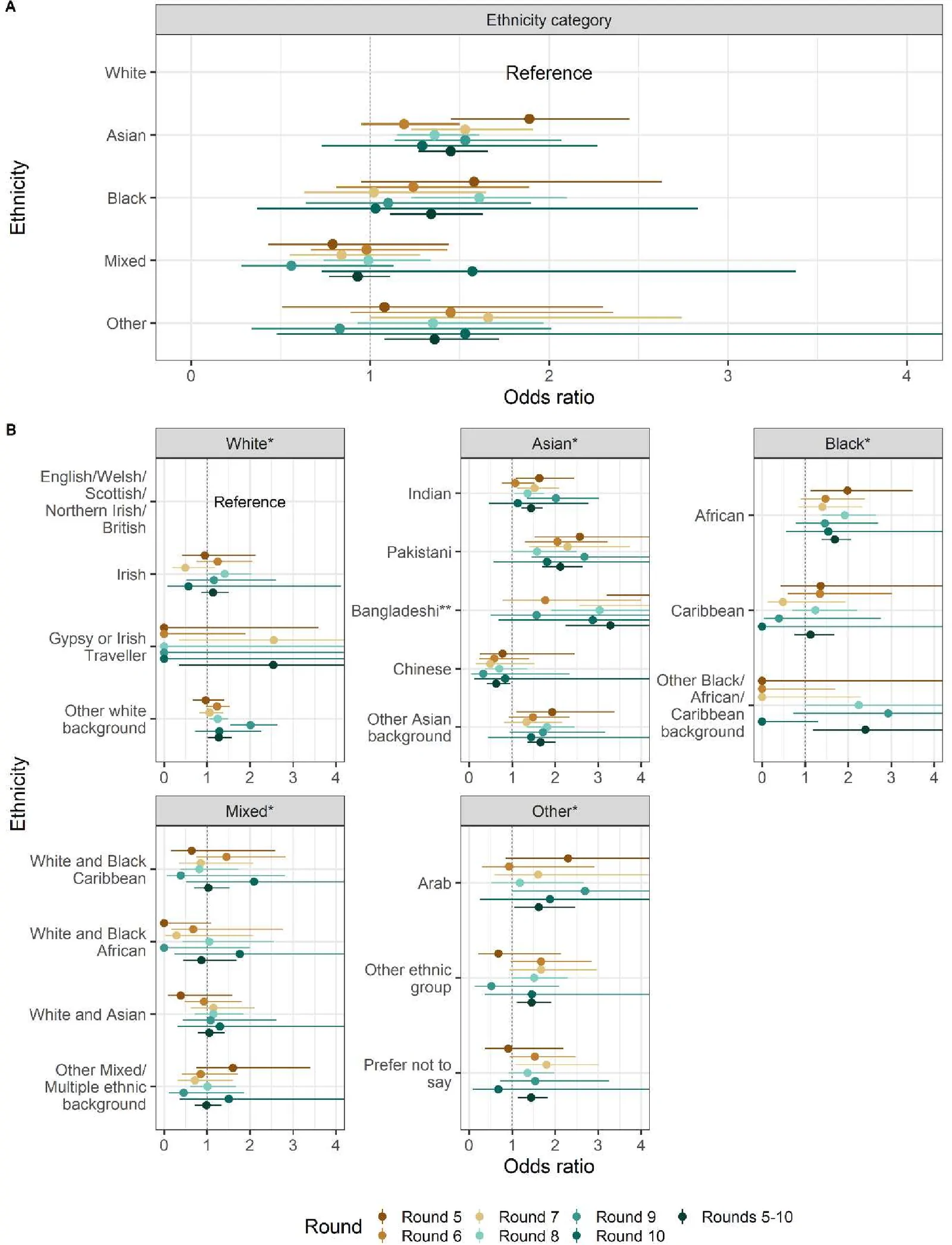
Forest plot of ethnicity categories and detailed subgroups of ethnicity for rounds 5–10 and an average across rounds weighted inverse to variance (rounds 5 to 10). (A) Forest plot of ethnicity categories, where the reference group is the White ethnicity. (B) Forest plot of detailed subgroups of ethnicity, the reference group is those identifying as English, Welsh, Scottish, Northern Irish or British. Individual round ORs are mutually adjusted for sex, age group, region, key worker status and deprivation index. Round 5: 18 September 2020 to 5 October 2020; Round 6: 16 October 2020 to 2 November 2020; Round 7: 13 November 2020 to 3 December 2020; Round 8: 6 January 2021 to 22 January 2021; Round 9: 4 February 2021 to 23 February 2021; Round 10: 11 March 2021 to 30 March 2021. *For detailed subgroups of ethnicity, they are included in one model where the reference group is those identifying as English, Welsh, Scottish, Northern Irish or British. **The OR for Bangladeshi ethnicity in round 5 was 5.17 (3.01,8.88).

Participants were also asked to self-identify as a member of an ethnic subgroup. Some self-identified ethnic subgroups had a higher prevalence of SARS-CoV-2 infection in some rounds and lower in others (online supplemental table S4). These temporal differences were substantial and likely reflected underlying epidemic dynamics,^42^ rather than chance variation. For example, from rounds 5–8, the prevalence of swab-positivity in Bangladeshi participants was very high, reaching 6.12% (3.78%, 9.29%) in round 8. This elevated prevalence could be linked to higher community transmission rates in areas with a higher concentration of Bangladeshi individuals or variations in adherence to social distancing measures. By comparison, the prevalence among the White ethnic subgroups was less than 2%, with the highest value found in the White Irish participants at 1.93% (1.30%, 2.74%). During the same period, the Black ethnic subgroups also showed a higher prevalence than the White ethnic subgroups. This pattern suggests that certain ethnic subgroups were more affected during specific phases of the epidemic, likely reflecting localised outbreak dynamics, differences in occupational exposure and socioeconomic factors that influence susceptibility to infection.

Excess risk of infection was also seen in these ethnic subgroups compared with the White British group, even after accounting for the differences in age, sex, region, employment status, household size and level of local neighbourhood deprivation. Among subgroups, across rounds 5–10, the highest and the second-highest odds were found in Bangladeshi and Pakistani ethnic subgroups at 3.28 (2.24, 4.8) and 2.12 (1.7, 2.64), respectively, compared with the White British participants, which contributed to the high odds in Asians (figure 2B, online supplemental table S5).

Our findings, based on detailed ethnicity, underscore the difference that exists within the broad Asian ethnic category. In contrast to Bangladeshi and Pakistani participants, those of Chinese descent showed no strong evidence of an increased risk of infection compared with the White British reference group; their overall OR across rounds 5–10 was near 1.0, with a 95% CI clearly overlapping the null effect (figure 2B).

### Household size and deprivation

Participants in larger households consistently had higher rates of infection than in smaller households. Prevalence increased monotonically with household size, and households with six or more people consistently had the highest prevalence in rounds 5 through 10. These results were maintained when estimating ORs adjusted for age and sex, where participants in households of 3–5 people and 6 or more people had overall increased odds of 1.30 (1.18, 1.43) and 1.92 (1.64, 2.24), respectively, compared with participants in households of size 1 or 2. This trend was accentuated by a model adjusted for region, key worker status, ethnicity and deprivation in addition to age and sex, with ORs of 1.32 (1.21, 1.44) and 1.80 (1.60, 2.03) respectively (online supplemental figure S1 and online supplemental table S3).

In rounds 5–10, participants in the most deprived areas consistently had a higher risk of infection than those in the least deprived areas, with an overall increased odds of 1.69 (1.51, 1.89) adjusted for age and sex. The deprivation difference remained after adjusting for age, sex, region, broad categories of employment type, broad categories of ethnicity and household size, with people in the most deprived quintile having an increased odds of 1.42 (1.23, 1.63) compared with the least deprived (figure 3, online supplemental table S3).

**Figure 3:**
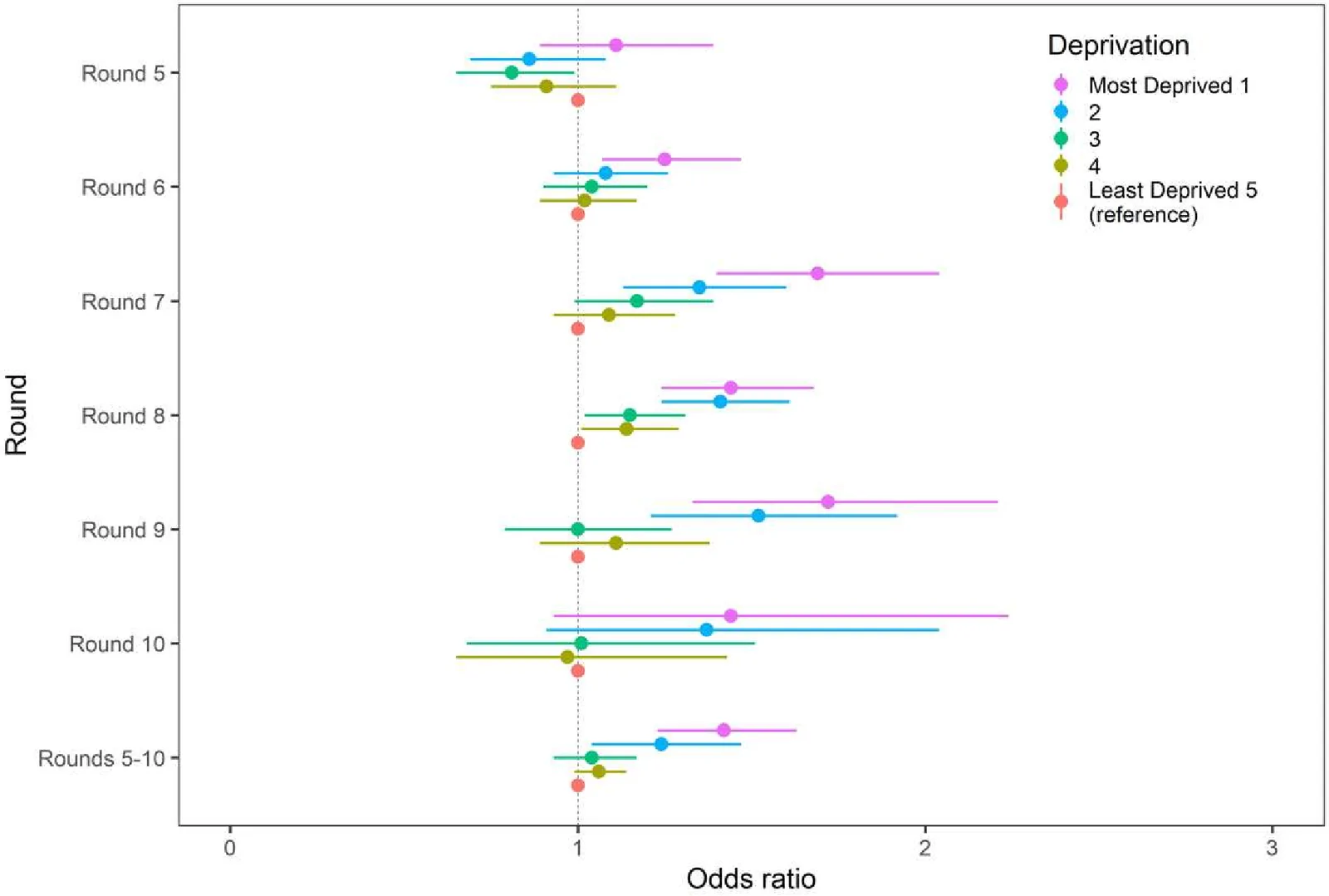
Forest plot of deprivation for rounds 5–10 and an average across rounds weighted inverse to variance (rounds 5–10). The reference group is those living in the least deprived areas (Least Deprived 5). Individual round ORs are mutually adjusted for sex, key worker status, broad categories of ethnicity, household size and deprivation index. Round 5: 18 September 2020 to 5 October 2020; Round 6: 16 October 2020 to 2 November 2020; Round 7: 13 November 2020 to 3 December 2020; Round 8: 6 January 2021 to 22 January 2021; Round 9: 4 February 2021 to 23 February 2021; Round 10: 11 March 2021 to 30 March 2021.

### Occupation

Occupation was also a risk factor for swab-positivity. Across rounds 5–10, compared with non-key workers, the odds of infections in healthcare and care home workers was 1.32 (1.13, 1.54) (online supplemental table S3). Among key workers, healthcare workers with direct patient contact had increased odds of 1.18 (1.04, 1.33), while the odds for healthcare workers with no patient contact were 0.77 (0.6, 0.99), compared with other essential/key workers. The odds of swab-positivity were increased among care home workers, both those with direct contact with clients at 1.35 (1.01, 1.81) and those without contact with clients at 2.44 (1.57, 3.79) compared with other essential/key workers (figure 4A, online supplemental tables 6 and 7).

**Figure 4:**
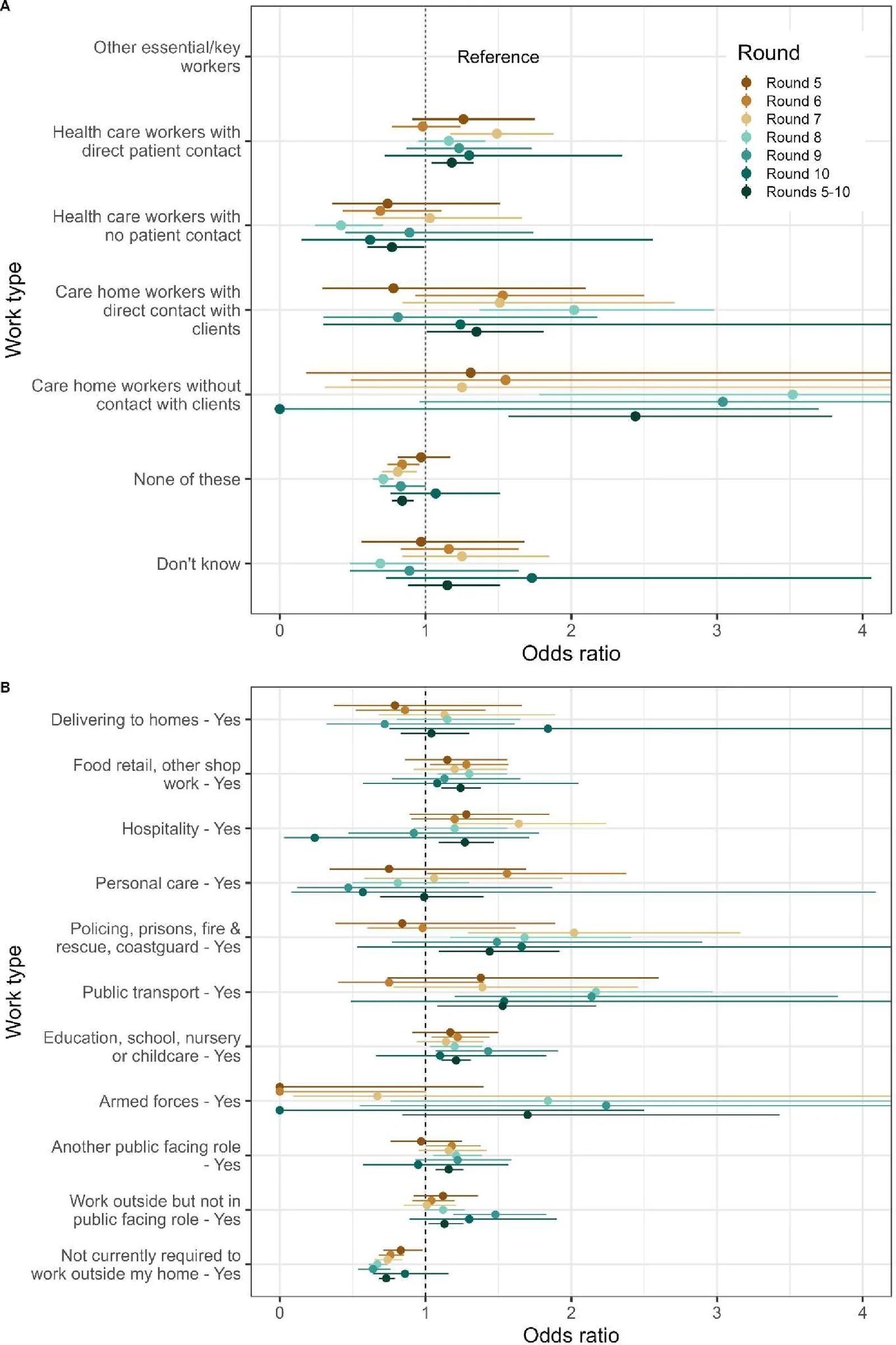
Forest plot of occupations for rounds 5–10 and an average across rounds weighted inverse to variance (rounds 5–10). (A) Reference group is other essential/key workers. Note that the bottom four categories are combined in (B) as HCW/CHW, and combined with full-time and part-time status in the variable named ‘key worker status’. (B) Only people who did not work in health or social care (top four boxes, Figure 4A) were asked whether their work involved these activities or settings. Each work activity or setting has a different reference group—those who answered that they did not work with that activity or in that setting. For example, the reference group for those that answered that they delivered to homes was the group who answered that they did not deliver to homes. Individual round ORs are mutually adjusted for sex, age group, region, broad category of ethnicity, deprivation index and the specific work activity or setting represented. Round 5: 18 September 2020 to 5 October 2020; Round 6: 16 October 2020 to 2 November 2020; Round 7: 13 November 2020 to 3 December 2020; Round 8: 6 January 2021 to 22 January 2021; Round 9: 4 February 2021 to 23 February 2021; Round 10: 11 March 2021 to 30 March 2021. HCW/CHW, health care worker/care home worker.

We also asked non-key workers about specific activities that they may or may not have had to undertake related to their work. For example, compared with those not occupationally exposed to the activity, participants whose work activity included: food retail or other shop work had increased odds of 1.24 (1.11, 1.38); hospitality had increased odds of 1.27 (1.09, 1.47); public transport had increased odds of 1.53 (1.08, 2.17); and participants working in education, school, nursery or childcare had increased odds of 1.21 (1.11, 1.31) (figure 4B, online supplemental table S7). The odds of infections for participants who were not required to work outside their homes were lower at 0.73 (0.68, 0.79) compared with those who were required to work outside their homes.

### Sensitivity analysis for vaccination status

We compared the results from the regression models reported above adjusted for sex, age, region, key worker status, ethnicity, household size and neighbourhood deprivation, with similar models also adjusted for vaccination status. The purpose of this sensitivity analysis was to determine whether the significant inequalities observed between different ethnicities and occupations persisted after accounting for vaccination status. There were no marked differences between these two sets of models (online supplemental table S8), indicating that the significant inequalities in infection risk between ethnic and occupational groups were not substantially affected by vaccination status. For example, the odds of SARS-CoV-2 infection remained higher among healthcare and care home workers at 1.87 (1.03, 3.38) after adjustment for vaccination status. Similarly, Asians continued to show a higher risk of infection before and after the adjustment. This suggests that the vaccination rates during this period were not sufficient to eliminate the disparities, and the underlying structural inequalities still need to be addressed.

Although we did observe participants who received one or two doses of vaccination had lower odds of infection than unvaccinated participants, with ORs of 0.63 (0.45, 0.86) and 0.61 (0.23, 1.59), respectively, these reductions in infection risk did not substantially alter the existing disparities between ethnic and occupational groups.

## Discussion

In this large community-based study of SARS-CoV-2 infection in England, we found during the period September 2020 to March 2021 that minority ethnic subgroups, larger household sizes, increasing neighbourhood deprivation and key worker status were independently associated with increased odds of testing positive for SARS-CoV-2. During that time, few people were vaccinated, and the perceived risk of infection was high. Our results suggest that substantial inequalities arose because of differential risk of infection rather than access to testing and healthcare.

Our findings build on prior descriptions of antigenic exposure to SARS-CoV-2 in a similar study in England during the period immediately prior to September 2020,^43^ which suggested that higher hospitalisation and deaths in ethnic minority subgroups were because of their higher infection risk, not because these groups were intrinsically more vulnerable to disease after infection.^44–48^ Therefore, the results presented here imply that health inequality driven by a higher risk of infection persisted in England through the second wave, during which there was high excess mortality.^49^

A 39-million-people study of health records in England by Larsen *et al*^50^ revealed the highest case rates in Bangladeshi and Pakistani ethnic groups, highlighting significant disparities in relative risk compared with the White British ethnic group during the second wave. However, it is important to note that the results from^50^ are not directly comparable to ours. The REACT-1 study, on which our research is based, is a cross-sectional study that randomly sampled individuals across LTLAs in England, regardless of current symptom status. This methodological difference means that while Larsen *et al*’s^50^ findings provide valuable context and evidence of disparities, they represent a different approach to data collection and analysis. In interpreting these results, it is also important to recognise the specificity of the national context of England. The high infection rates observed in the UK’s Bangladeshi and Pakistani communities are understood to be driven by well-documented structural inequalities, including overrepresentation in frontline occupations and higher prevalence of overcrowded, multigenerational housing.^48,51,52^ This national context is important, as the same ethnic group may have a different socioeconomic profile in other countries. The study by Chilunga *et al*^53^ investigates health inequalities among populations with a migration background in the Netherlands and differs in context and focus from our research using the REACT-1 study in the UK. However, it raises an important point about potential parallels in health inequalities across countries with similar demographic structures for minority groups. Although our study concentrates on different ethnic groups and activities within the UK, our findings are consistent with those of Chilunga *et al*^53^ in highlighting a broader pattern of disparities. In particular, both studies report that minority ethnic populations experienced a disproportionate burden during the pandemic, including elevated infection risk, restricted access to protective resources and increased socioeconomic vulnerability. These observations point to underlying structural factors that produce similar patterns of inequity across diverse national contexts.

Factors driving increased infection in ethnic minorities are not immediately obvious: for example, one study of social mixing patterns during 2020^54^ found reduced rates of mixing in some ethnic groups, rather than increased rates, which would provide a mechanism for increased rates of infection. This paradox highlights that standard social mixing metrics typically capture discretionary contacts while failing to account for key non-discretionary and structural exposure routes. A critical cultural and socioeconomic factor is household composition. In the UK, older adults of South Asian background (particularly those of Bangladeshi and Pakistani ethnicity) are significantly more likely to live in multigenerational households compared with their White counterparts.^55^ This household structure increases the baseline risk of household transmission from members who cannot work from home.

Therefore, structural factors such as occupation provide a more related and direct explanation. Multiple longitudinal studies found significant occupational inequalities in COVID-19 prevalence during a similar time period to our study, reinforcing that workplace exposure is a key determinant of infection risk, particularly for roles that cannot be done from home.^56,57^ However, this may have been driven by response bias during a period when many people were at home with more time to enrol in surveys compared with those going out to work.^58^ Recruitment bias may also explain findings from a US study which analysed mobility data as a proxy for mixing, using variation in the ethnic diversity in each county to infer relative levels of mixing for different ethnic groups.^59^ They found that African Americans had the lowest reduction in mobility and the highest rates of severe disease compared with White and Asian American populations. It is plausible that such mobility patterns are linked to occupational requirements rather than social mixing alone.

These results represent substantial evidence that variation in infection drives variation in disease among ethnic groups. While other studies have speculated that other factors explain differences in patterns of disease, such as genetic susceptibility,^60–62^ treatment access and comorbidities,^63^ such as obesity,^64^ diabetes^65^ and hypertension,^66^ few studies fully capture their degree of representativeness or measure infection, making it difficult to identify the root causes of increased disease. Further studies of this design are warranted to better characterise the contribution of these different factors to health inequality arising from infectious disease.^67^

Our study has limitations. First, we aimed to obtain representative samples for the population of England. Although we were able to correct for variations in response and estimate weighted prevalence for the population of England and specific subgroups, the number of people taking part in some subgroups was relatively low; this resulted in higher uncertainty in the estimates (with wider 95% CIs). For example, we chose to present weighted prevalence only if the number of positives in a category was 10 or more; among people of ethnic minorities during some periods, the number of positives was too small to obtain robust estimates for weighted prevalence using classic survey-weighting methods. As a result, some important differences between subgroups were not fully captured. Although other analytical approaches, such as Bayesian hierarchical regression, may be better able to infer patterns where the sample size is small,^68^ they would also require additional assumptions about the relationship between hierarchies, which we decided were not justified.

We considered modelling interaction effects between sociodemographic variables in order to more directly examine intersectionality. However, for several subgroups, particularly when stratified jointly by ethnicity, region and occupation, the sample sizes were insufficient to produce stable estimates. In many intersectional strata, fewer than 10 individuals were observed, especially among ethnic minority populations, which introduced substantial uncertainty and a risk of biased or uninterpretable results. While future studies leveraging larger or pooled datasets may be better positioned to investigate these complex interactions, our focus in this study remained on main effects, given the limitations imposed by sample sparsity.

The effects of risk factors might vary over time, for example, due to changes in contact or behavioural patterns among subgroups. Thus, students returning to universities from home after the holiday will increase contacts while travelling and on arrival at university. In addition, as the vaccine programme in England prioritised certain subgroups of the population, they might have acquired sufficient immunity to reduce the risk of infections^69^ in ways not captured by our sensitivity analyses. Although we investigated ORs for risk factors by rounds, we did not investigate within-round temporal trends in the relationship between risk factors and swab positivity.

Furthermore, response rates were lower for minority ethnic groups. In theory, this could have biased these results. However, we would expect such bias to have decreased rates of infection in these groups rather than increased them, as people who take part in surveys tend to have lower rates of exposure.^70^ Additionally, being present at home during lockdown periods made participation in our study easier and less likely to have been in contact with other people.^71,72^ This limitation highlights the potential for even greater disparities in infection risk among minority ethnic groups than those observed in our study.

Our study is cross-sectional, which precludes the longitudinal tracking of individuals across successive pandemic waves. Therefore, we could not determine whether participants with a high risk of infection in the second wave, such as those from minoritised ethnic groups, had also been infected during the first wave. Understanding the role of naturally acquired immunity and the potential for reinfection is critical for accurately modelling pandemic dynamics and health inequalities over time, and can be addressed using studies with a longitudinal design.^73,74^ These issues should be considered when designing cohort studies for future outbreaks of pandemics of viral respiratory infections.

Our analysis focused primarily on exposure-related variables, including occupational classification and household size, which are widely recognised predictors of SARS-CoV-2 infection risk. However, we acknowledge that additional variables related to behavioural exposure and individual susceptibility were not included in the current model. Examples include whether children in the household attended school,^75^ the reasons for and frequency of leaving home,^76^ the use of face coverings^77^ and the presence of pre-existing health conditions such as immunosuppression.^76^ These factors may influence infection risk and could act as confounders. Future work should consider incorporating a broader set of behavioural and susceptibility-related variables to provide a more comprehensive understanding of the mechanisms driving infection disparities across population subgroups.

In conclusion, our findings show substantial differences in the risk of SARS-CoV-2 infections among minority ethnic groups and other subgroups of the population in England during a period when there was a substantial risk of disease and death from COVID-19. Planning for future severe waves of respiratory pathogens should include policies to reduce inequalities driven by ethnicity, household size and occupational activity.^78^ Such policies could involve enhancing workplace protections, providing support for overcrowded households, ensuring equitable access to healthcare and implementing targeted public health campaigns. Additionally, improving access to vaccination through outreach programmes, offering economic support and job security for high-risk workers, and engaging communities in decision-making processes are crucial steps. These measures can help mitigate the disparities in infection risk and outcomes, contributing to a more equitable public health response.

## Supplementary material

10.1136/bmjph-2025-002596 online supplemental figure 1

10.1136/bmjph-2025-002596 online supplemental table 1

10.1136/bmjph-2025-002596 online supplemental table 2

10.1136/bmjph-2025-002596 online supplemental table 3

10.1136/bmjph-2025-002596 Supplementary Table 4

10.1136/bmjph-2025-002596 Supplementary Table 5

10.1136/bmjph-2025-002596 online supplemental Table 6

10.1136/bmjph-2025-002596 online supplemental table 7

10.1136/bmjph-2025-002596 online supplemental table 8

## Data Availability

Data are available upon reasonable request.
