# Supplementary material for: Health inequalities in risk of SARS-CoV-2 infection in England’s second wave: population-based cross-sectional analysis from the REACT-1 study
Source: BMJ Public Health. 2026 Jan 5;4(1):e002596. doi: 10.1136/bmjph-2025-002596 (PMC13663695; doi:10.1136/bmjph-2025-002596)
Supplement: Supplementary file 1 [file bmjph-4-1-s001.pdf]

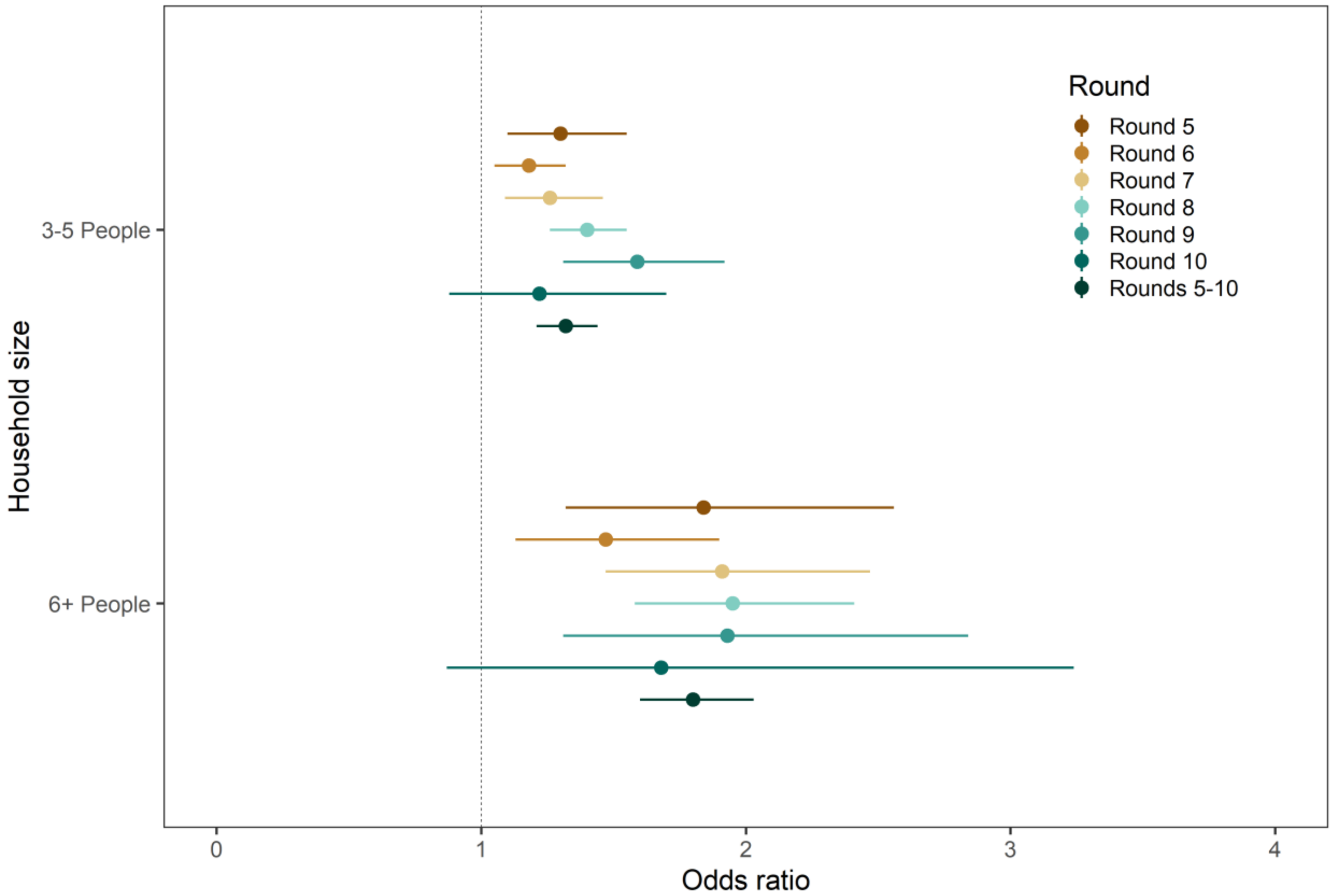


**Supplementary Figure 1.** Forest plot of household size for rounds 5 to 10 and an average weighted across rounds inverse to variance (rounds 5 to 10). The reference group is not shown in the figure and is households with 1-2 people. Individual round odds ratios are mutually adjusted for sex, key worker status, broad categories of ethnicity, household size and neighbourhood deprivation.

Round 5: 18/09/2020 to 05/10/2020

Round 6: 16/10/2020 to 02/11/2020

Round 7: 13/11/2020 to 03/12/2020

Round 8: 06/01/2021 to 22/01/2021

Round 9: 04/02/2021 to 23/02/2021

Round 10: 11/03/2021 to 30/03/2021
