# Supplementary material for: Health inequalities in risk of SARS-CoV-2 infection in England’s second wave: population-based cross-sectional analysis from the REACT-1 study
Source: BMJ Public Health. 2026 Jan 5;4(1):e002596. doi: 10.1136/bmjph-2025-002596 (PMC13663695; doi:10.1136/bmjph-2025-002596)
Supplement: Supplementary file 2 [file bmjph-4-1-s002.pdf]

Supplementary Table 1. The unweighted and weighted prevalence of swab-positivity across rounds 5 to 10 of REACT-1.

Round*	Tested swabs	Positive swabs	Unweighted prevalence (95% CI)	Weighted prevalence (95% CI)	First sample	Last sample
5	174,949	824	0.47% (0.44%, 0.50%)	0.60% (0.55%, 0.71%)	18/09/2020	05/10/2020
6	160,173	1,731	1.08% (1.03%, 1.13%)	1.30% (1.21%, 1.39%)	16/10/2020	02/11/2020
7	168,181	1,299	0.77% (0.73%, 0.82%)	0.94% (0.87%, 1.01%)	13/11/2020	03/12/2020
8	167,642	2,282	1.36% (1.31%, 1.42%)	1.57% (1.49%, 1.66%)	06/01/2021**	22/01/2021
9	165,456	689	0.42% (0.39%, 0.45%)	0.49% (0.44%, 0.55%)	04/02/2021	23/02/2021
10	140,844	227	0.16% (0.14%, 0.18%)	0.20% (0.17%, 0.23%)	11/03/2021	30/03/2021

* Rounds 1 to 4 were conducted between 1/5/2020 and 8/9/2020 and found 473 positives from 596,565 tested swabs

* Includes small number of samples from previous days
