# Supplementary material for: Health inequalities in risk of SARS-CoV-2 infection in England’s second wave: population-based cross-sectional analysis from the REACT-1 study
Source: BMJ Public Health. 2026 Jan 5;4(1):e002596. doi: 10.1136/bmjph-2025-002596 (PMC13663695; doi:10.1136/bmjph-2025-002596)
Supplement: Supplementary file 3 [file bmjph-4-1-s003.pdf]

Supplementary Table 2. The unweighted and weighted prevalence of swab-positivity by socio-demographic variables for rounds 5 to 10.

Variable	Category	Round 5				Round 6				Round 7				Round 8			
		Positive	Total	Unweighted Prevalence	Weighted Prevalence*	Positive	Total	Unweighted Prevalence	Weighted Prevalence*	Positive	Total	Unweighted Prevalence	Weighted Prevalence*	Positive	Total	Unweighted Prevalence	Weighted Prevalence*
Gender	Male	387	78,564	0.49% (0.44% , 0.54%)	0.65% (0.57% , 0.75%)	769	72,315	1.06% (0.99% , 1.14%)	1.29% (1.17% , 1.43%)	599	75,656	0.79% (0.73% , 0.86%)	0.96% (0.86% , 1.07%)	1043	75,296	1.39% (1.30% , 1.47%)	1.63% (1.50% , 1.77%)
	Female	437	96,380	0.45% (0.41% , 0.50%)	0.54% (0.48% , 0.62%)	962	87,852	1.10% (1.03% , 1.17%)	1.31% (1.19% , 1.44%)	700	92,524	0.76% (0.70% , 0.81%)	0.92% (0.83% , 1.02%)	1239	92,340	1.34% (1.27% , 1.42%)	1.51% (1.41% , 1.63%)
	NA	0	4	0.00% (0.00% , 60.24%)	NA (NA , NA)	0	6	0.00% (0.00% , 45.93%)	NA (NA , NA)	0	1	0.00% (0.00% , 97.50%)	NA (NA , NA)	0	6	0.00% (0.00% , 31.08%)	NA (NA , NA)
Age	05-12	59	13,771	0.43% (0.33% , 0.55%)	0.58% (0.47% , 0.73%)	113	11,245	1.00% (0.83% , 1.21%)	1.27% (0.96% , 1.68%)	147	11,873	1.24% (1.05% , 1.45%)	1.44% (1.16% , 1.77%)	170	11,727	1.45% (1.25% , 1.68%)	1.59% (1.32% , 1.93%)
	13-17	59	10,480	0.56% (0.43% , 0.73%)	0.60% (0.43% , 0.84%)	119	8,616	1.38% (1.15% , 1.65%)	1.72% (1.33% , 2.21%)	172	9,861	1.74% (1.50% , 2.02%)	2.04% (1.69% , 2.46%)	172	8,947	1.92% (1.66% , 2.23%)	2.25% (1.85% , 2.73%)
	18-24	83	6,852	1.21% (0.97% , 1.50%)	0.70% (0.49% , 1.00%)	120	6,308	1.90% (1.58% , 2.27%)	2.38% (1.79% , 3.16%)	86	6,475	1.33% (1.06% , 1.64%)	1.41% (1.04% , 1.91%)	156	6,753	2.31% (1.98% , 2.70%)	2.44% (1.96% , 3.03%)
	25-34	86	15,683	0.55% (0.44% , 0.68%)	1.59% (1.18% , 2.13%)	206	15,242	1.35% (1.17% , 1.55%)	1.56% (1.30% , 1.86%)	130	14,758	0.88% (0.74% , 1.05%)	1.00% (0.80% , 1.23%)	263	15,031	1.75% (1.55% , 1.97%)	1.95% (1.68% , 2.26%)
	35-44	121	22,954	0.53% (0.44% , 0.63%)	0.69% (0.53% , 0.90%)	214	21,003	1.02% (0.89% , 1.16%)	1.25% (1.05% , 1.48%)	160	20,846	0.77% (0.65% , 0.90%)	0.89% (0.73% , 1.09%)	314	21,764	1.44% (1.29% , 1.61%)	1.62% (1.41% , 1.85%)
	45-54	157	29,380	0.53% (0.45% , 0.62%)	0.58% (0.48% , 0.70%)	313	26,897	1.16% (1.04% , 1.30%)	1.26% (1.10% , 1.44%)	220	27,338	0.80% (0.70% , 0.92%)	0.86% (0.73% , 1.01%)	412	27,982	1.47% (1.34% , 1.62%)	1.61% (1.43% , 1.81%)
	55-64	116	31,884	0.36% (0.30% , 0.44%)	0.37% (0.30% , 0.46%)	326	29,752	1.10% (0.98% , 1.22%)	1.20% (1.05% , 1.38%)	188	31,396	0.60% (0.52% , 0.69%)	0.64% (0.53% , 0.76%)	413	31,989	1.29% (1.17% , 1.42%)	1.29% (1.15% , 1.45%)
	65+	143	43,944	0.33% (0.27% , 0.38%)	0.35% (0.28% , 0.43%)	320	41,110	0.78% (0.70% , 0.87%)	0.82% (0.71% , 0.94%)	196	45,634	0.43% (0.37% , 0.49%)	0.50% (0.41% , 0.61%)	382	43,449	0.88% (0.80% , 0.97%)	0.93% (0.82% , 1.05%)
Region	South East	104	39,496	0.26% (0.22% , 0.32%)	0.29% (0.23% , 0.37%)	244	36,164	0.67% (0.59% , 0.76%)	0.69% (0.60% , 0.80%)	254	38,296	0.66% (0.58% , 0.75%)	0.73% (0.63% , 0.84%)	578	39,156	1.48% (1.36% , 1.60%)	1.61% (1.46% , 1.77%)
	North East	61	6,702	0.91% (0.70% , 1.17%)	1.06% (0.80% , 1.42%)	89	5,846	1.52% (1.22% , 1.87%)	1.49% (1.17% , 1.89%)	53	5,673	0.93% (0.70% , 1.22%)	0.94% (0.66% , 1.35%)	62	5,731	1.08% (0.84% , 1.38%)	1.22% (0.87% , 1.69%)
	North West	199	19,468	1.02% (0.89% , 1.17%)	1.21% (1.01% , 1.46%)	372	17,866	2.08% (1.88% , 2.30%)	2.39% (2.10% , 2.73%)	157	18,337	0.86% (0.73% , 1.00%)	1.02% (0.83% , 1.24%)	220	17,917	1.23% (1.08% , 1.40%)	1.38% (1.16% , 1.64%)
	Yorkshire and The Humber	74	11,658	0.63% (0.50% , 0.80%)	0.84% (0.60% , 1.17%)	189	10,697	1.77% (1.53% , 2.03%)	2.32% (1.90% , 2.81%)	107	11,288	0.95% (0.78% , 1.14%)	1.25% (0.99% , 1.58%)	93	10,665	0.87% (0.71% , 1.07%)	0.80% (0.62% , 1.03%)
	East Midlands	101	21,897	0.46% (0.38% , 0.56%)	0.56% (0.43% , 0.72%)	233	20,458	1.14% (1.00% , 1.29%)	1.25% (1.07% , 1.45%)	221	22,001	1.00% (0.88% , 1.15%)	1.19% (1.00% , 1.41%)	225	21,698	1.04% (0.91% , 1.18%)	1.16% (0.99% , 1.36%)
	West Midlands	70	16,343	0.43% (0.33% , 0.54%)	0.60% (0.40% , 0.89%)	196	14,860	1.32% (1.14% , 1.52%)	1.59% (1.18% , 2.13%)	156	15,757	0.99% (0.84% , 1.16%)	1.24% (0.95% , 1.61%)	198	15,088	1.31% (1.14% , 1.51%)	1.66% (1.32% , 2.10%)
	East of England	81	25,625	0.32% (0.25% , 0.39%)	0.30% (0.23% , 0.38%)	150	23,231	0.65% (0.55% , 0.76%)	0.69% (0.57% , 0.83%)	125	24,627	0.51% (0.42% , 0.60%)	0.58% (0.47% , 0.71%)	391	25,178	1.55% (1.41% , 1.71%)	1.78% (1.57% , 2.02%)
	London	79	17,499	0.45% (0.36% , 0.56%)	0.54% (0.42% , 0.70%)	141	15,757	0.89% (0.75% , 1.05%)	0.97% (0.79% , 1.18%)	136	15,566	0.87% (0.73% , 1.03%)	1.09% (0.89% , 1.32%)	390	15,641	2.49% (2.26% , 2.75%)	2.83% (2.53% , 3.16%)
	South West	55	16,260	0.34% (0.25% , 0.44%)	0.33% (0.23% , 0.46%)	117	15,294	0.77% (0.63% , 0.92%)	0.80% (0.64% , 0.99%)	90	16,636	0.54% (0.44% , 0.66%)	0.58% (0.45% , 0.76%)	125	16,568	0.75% (0.63% , 0.90%)	0.87% (0.71% , 1.08%)
key worker status	Health care or care home worker	51	9,620	0.53% (0.40% , 0.70%)	0.56% (0.48% , 0.65%)	124	9,257	1.34% (1.12% , 1.60%)	1.78% (1.33% , 2.39%)	122	9,460	1.29% (1.07% , 1.54%)	1.54% (1.23% , 1.94%)	161	8,259	1.95% (1.67% , 2.27%)	2.24% (1.84% , 2.72%)
	Other essential/key worker	154	28,846	0.53% (0.45% , 0.62%)	0.66% (0.46% , 0.94%)	357	26,894	1.33% (1.19% , 1.47%)	1.64% (1.41% , 1.92%)	267	29,910	0.89% (0.79% , 1.01%)	1.04% (0.88% , 1.22%)	531	30,239	1.76% (1.61% , 1.91%)	1.79% (1.61% , 2.00%)
	Other workers	338	72,174	0.47% (0.42% , 0.52%)	0.70% (0.55% , 0.88%)	677	64,810	1.04% (0.97% , 1.13%)	1.22% (1.10% , 1.37%)	457	64,423	0.71% (0.65% , 0.78%)	0.82% (0.72% , 0.93%)	867	64,772	1.34% (1.25% , 1.43%)	1.55% (1.42% , 1.70%)
	Not full-time, part-time, or self-employed	256	59,762	0.43% (0.38% , 0.48%)	0.58% (0.49% , 0.69%)	519	55,444	0.94% (0.86% , 1.02%)	1.11% (0.98% , 1.25%)	380	60,222	0.63% (0.57% , 0.70%)	0.85% (0.74% , 0.97%)	670	60,438	1.11% (1.03% , 1.20%)	1.36% (1.23% , 1.50%)
	NA	25	4,546	0.55% (0.36% , 0.81%)	0.60% (0.37% , 0.95%)	54	3,768	1.43% 1.08% 1.87%	1.55% (1.04% , 2.31%)	73	4,166	1.75% (1.38% , 2.20%)	1.80% (1.34% , 2.41%)	53	3,934	1.35% (1.03% , 1.76%)	1.58% (1.10% , 2.27%)
Ethnic group	White	707	157,644	0.45% (0.42% , 0.48%)	0.54% (0.48% , 0.59%)	1549	145,334	1.07% (1.01% , 1.12%)	1.24% (1.16% , 1.33%)	1122	153,463	0.73% (0.69% , 0.78%)	0.84% (0.78% , 0.90%)	1938	152,111	1.27% (1.22% , 1.33%)	1.41% (1.33% , 1.49%)
	Asian	71	7,907	0.90% (0.70% , 1.13%)	1.11% (0.83% , 1.48%)	84	6,712	1.25% (1.00% , 1.55%)	2.05% (1.45% , 2.88%)	95	6,577	1.44% (1.17% , 1.76%)	2.07% (1.57% , 2.71%)	163	6,537	2.49% (2.14% , 2.90%)	2.80% (2.32% , 3.37%)
	Black	16	2,194	0.73% (0.42% , 1.18%)	1.17% (0.65% , 2.10%)	23	1,760	1.31% (0.83% , 1.95%)	1.23% (0.76% , 1.98%)	18	1,754	1.03% (0.61% , 1.62%)	1.07% (0.65% , 1.76%)	60	1,832	3.28% (2.55% , 4.19%)	3.07% (2.29% , 4.09%)
	Mixed	11	3,080	0.36% (0.18% , 0.64%)	0.44% (0.22% , 0.86%)	28	2,697	1.04% (0.69% , 1.50%)	1.01% (0.66% , 1.53%)	23	2,721	0.85% (0.54% , 1.27%)	0.84% (0.53% , 1.33%)	46	2,751	1.67% (1.26% , 2.22%)	1.78% (1.28% , 2.46%)
	Other	7	1,439	0.49% (0.20% , 1.00%)	0.47% (0.21% , 1.05%)	17	1,184	1.44% (0.84% , 2.29%)	1.81% (1.00% , 3.24%)	16	1,157	1.38% (0.79% , 2.24%)	1.97% (1.09% , 3.51%)	29	1,190	2.44% (1.70% , 3.48%)	2.42% (1.59% , 3.65%)
	NA	12	2,684	0.45% 0.23% 0.78%	0.68% (0.29% , 1.57%)	30	2,486	1.21% 0.82% 1.72%	1.38% (0.87% , 2.18%)	25	2,509	1.00% (0.65% , 1.47%)	1.15% (0.74% , 1.78%)	46	3,221	1.43% 1.07% 1.90%	1.76% (1.23% , 2.50%)
Household size	1	88	24,916	0.35% (0.28% , 0.44%)	0.44% (0.32% , 0.59%)	228	23,639	0.96% (0.84% , 1.10%)	1.08% (0.91% , 1.29%)	125	25,606	0.49% (0.41% , 0.58%)	0.56% (0.44% , 0.71%)	275	25,360	1.08% (0.96% , 1.22%)	1.24% (1.06% , 1.44%)
	2	227	61,717	0.37% (0.32% , 0.42%)	0.44% (0.37% , 0.53%)	553	59,221	0.93% (0.86% , 1.01%)	1.13% (0.99% , 1.28%)	351	62,446	0.56% (0.51% , 0.62%)	0.69% (0.60% , 0.79%)	622	61,379	1.01% (0.94% , 1.10%)	1.11% (1.01% , 1.23%)
	3	172	32,394	0.53% (0.45% , 0.62%)	0.61% (0.51% , 0.74%)	358	30,041	1.19% (1.07% , 1.32%)	1.47% (1.25% , 1.73%)	277	30,729	0.90% (0.80% , 1.01%)	0.96% (0.83% , 1.12%)	455	29,965	1.52% (1.39% , 1.66%)	1.67% (1.49% , 1.88%)
	4	211	37,231	0.57% (0.49% , 0.65%)	0.75% (0.61% , 0.92%)	349	31,911	1.09% (0.98% , 1.21%)	1.30% (1.11% , 1.52%)	315	33,498	0.94% (0.84% , 1.05%)	1.02% (0.89% , 1.17%)	568	34,847	1.63% (1.50% , 1.77%)	1.77% (1.59% , 1.96%)
	5	77	13,145	0.59% (0.46% , 0.73%)	0.76% (0.56% , 1.03%)	172	10,795	1.59% (1.37% , 1.85%)	1.73% (1.44% , 2.08%)	147	11,274	1.30% (1.10% , 1.53%)	1.59% (1.27% , 1.98%)	246	11,566	2.13% (1.88% , 2.41%)	2.48% (2.05% , 3.00%)
	6 and 6+	49	5,545	0.88% (0.65% , 1.17%)	1.05% (0.75% , 1.47%)	71	4,566	1.56% (1.22% , 1.96%)	2.16% (1.49% , 3.12%)	84	4,628	1.82% (1.45% , 2.24%)	2.40% (1.71% , 3.35%)	116	4,525	2.56% (2.14% , 3.07%)	3.01% (2.39% , 3.77%)
COVID case contac	No	556	139,853	0.40% (0.37% , 0.43%)	0.50% (0.44% , 0.56%)	1130	128,069	0.88% (0.83% , 0.94%)	1.02% (0.94% , 1.11%)	705	133,663	0.53% (0.49% , 0.57%)	0.65% (0.59% , 0.71%)	1130	138,065	0.82% (0.77% , 0.87%)	0.94% (0.87% , 1.02%)
	Yes, contact with a confirmed/tested COVID-19 case	102	1,566	6.51% (5.34% , 7.85%)	8.20% (6.31% , 10.59%)	301	3,621	8.31% (7.43% , 9.26%)	8.92% (7.67% , 10.35%)	362	4,658	7.77% (7.02% , 8.58%)	7.77% (6.83% , 8.83%)	730	5,008	14.58% (13.63% , 15.58%)	15.61% (14.26% , 17.06%)
	Yes, contact with a suspected COVID-19 case	17	1,487	1.14% (0.67% , 1.82%)	1.09% (0.64% , 1.83%)	50	1,669	3.00% (2.23% , 3.93%)	3.55% (2.26% , 5.53%)	31	1,495	2.07% (1.41% , 2.93%)	1.96% (1.27% , 3.02%)	58	1,329	4.36% (3.39% , 5.60%)	4.94% (3.57% , 6.79%)
	NA	149	32,042	0.47% (0.39% , 0.55%)	0.54% (0.43% , 0.68%)	250	26,814	0.93% (0.82% , 1.05%)	1.20% (0.99% , 1.45%)	201	28,365	0.71% (0.61% , 0.81%)	0.92% (0.75% , 1.14%)	364	23,240	1.57% (1.41% , 1.73%)	1.64% (1.45% , 1.87%)
Symptom status	Classic COVID	187	8,376	2.23% (1.93% , 2.57%)	2.75% (2.25% , 3.36%)	422	6,283	6.72% (6.11% , 7.36%)	7.51% (6.63% , 8.49%)	404	5,280	7.65% (6.95% , 8.40%)	7.98% (7.07% , 9.01%)	822	6,475	12.70% (11.91% , 13.53%)	14.05% (12.89% , 15.30%)
	Other symptoms	146	25,832	0.57% (0.48% , 0.66%)	0.70% (0.56% , 0.87%)	266	20,739	1.28% (1.13% , 1.45%)	1.35% (1.17% , 1.57%)	220	18,185	1.21% (1.06% , 1.38%)	1.35% (1.14% , 1.60%)	350	19,609	1.78% (1.61% , 1.98%)	1.89% (1.66% , 2.15%)
	No symptoms	344	108,910	0.32% (0.28% , 0.35%)	0.39% (0.34% , 0.46%)	798	106,439	0.75% (0.70% , 0.80%)	0.90% (0.81% , 1.01%)	476	116,449	0.41% (0.37% , 0.45%)	0.51% (0.46% , 0.58%)	747	118,401	0.63% (0.59% , 0.68%)	0.76% (0.68% , 0.84%)
	NA	147	31,830	0.46% (0.39% , 0.54%)	0.54% (0.43% , 0.68%)	245	26,712	0.92% (0.81% , 1.04%)	1.19% (0.98% , 1.45%)	199	28,267	0.70% (0.61% , 0.81%)	0.92% (0.74% , 1.14%)	363	23,157	1.57% (1.42% , 1.74%)	1.65% (1.45% , 1.87%)
Deprivation	1 Most deprived	135	17,641	0.77% (0.64% , 0.91%)	1.00% (0.80% , 1.26%)	254	15,736	1.61% (1.42% , 1.82%)	2.09% (1.74% , 2.50%)	198	15,951	1.24% (1.08% , 1.43%)	1.41% (1.19% , 1.67%)	257	15,190	1.69% (1.50% , 1.91%)	1.79% (1.53% , 2.10%)
	2	129	27,483	0.47% (0.39% , 0.56%)	0.53% (0.43% , 0.65%)	289	24,957	1.16% (1.03% , 1.30%)	1.27% (1.11% , 1.46%)	238	26,259	0.91% (0.80% , 1.03%)	1.04% (0.86% , 1.25%)	449	26,012	1.73% (1.57% , 1.89%)	1.95% (1.74% , 2.19%)
	3	146	37,426	0.39% (0.33% , 0.46%)	0.48% (0.38% , 0.59%)	354	34,378	1.03% (0.93% , 1.14%)	1.09% (0.96% , 1.24%)	269	35,975	0.75% (0.66% , 0.84%)	0.80% (0.69% , 0.93%)	479	36,061	1.33% (1.22% , 1.45%)	1.52% (1.36% , 1.69%)
	4	190	43,109	0.44% (0.38% , 0.51%)	0.49% (0.41% , 0.58%)	407	39,792	1.02% (0.93% , 1.13%)	1.10% (0.98% , 1.25%)	293	42,350	0.69% (0.62% , 0.78%)	0.83% (0.71% , 0.95%)	540	42,002	1.29% (1.18% , 1.40%)	1.41% (1.27% , 1.56%)
	5 Least deprived	224	49,289	0.45% (0.40% , 0.52%)	0.52% (0.44% , 0.61%)	427	45,310	0.94% (0.86% , 1.04%)	1.02% (0.91% , 1.15%)	301	47,646	0.63% (0.56% , 0.71%)	0.68% (0.59% , 0.78%)	557	48,377	1.15% (1.06% , 1.25%)	1.22% (1.10% , 1.35%)

* We present weighted prevalence if the number of positives in a category is 10 or more.

Round 9				Round 10			
Positive	Total	Unweighted Prevalence	Weighted Prevalence*	Positive	Total	Unweighted Prevalence	Weighted Prevalence*
315	75,027	0.42% (0.38% , 0.47%)	0.50% (0.43% , 0.59%)	108	63,077	0.17% (0.14% , 0.21%)	0.22% (0.17% , 0.28%)
374	90,425	0.41% (0.37% , 0.46%)	0.49% (0.43% , 0.56%)	119	77,765	0.15% (0.13% , 0.18%)	0.18% (0.14% , 0.22%)
0	4	0.00% (0.00% , 40.35%)	NA (NA , NA)	0	2	0 (0 , 84.19%)	NA (NA , NA)
81	13,605	0.60% (0.48% , 0.74%)	0.72% (0.54% , 0.95%)	33	10,750	0.31% (0.21% , 0.43%)	0.41% (0.27% , 0.62%)
45	10,155	0.44% (0.33% , 0.59%)	0.57% (0.38% , 0.87%)	14	7,891	0.18% (0.10% , 0.30%)	0.19% (0.11% , 0.34%)
40	6,788	0.59% (0.43% , 0.80%)	0.71% (0.45% , 1.12%)	13	5,799	0.22% (0.12% , 0.38%)	0.29% (0.16% , 0.53%)
68	14,801	0.46% (0.36% , 0.58%)	0.46% (0.34% , 0.61%)	21	12,203	0.17% (0.11% , 0.26%)	0.18% (0.11% , 0.31%)
94	21,088	0.45% (0.36% , 0.55%)	0.57% (0.42% , 0.77%)	35	17,339	0.20% (0.14% , 0.28%)	0.21% (0.14% , 0.31%)
132	27,595	0.48% (0.40% , 0.57%)	0.50% (0.41% , 0.62%)	37	22,258	0.17% (0.12% , 0.23%)	0.20% (0.13% , 0.30%)
135	31,727	0.43% (0.36% , 0.50%)	0.49% (0.40% , 0.60%)	39	25,811	0.15% (0.11% , 0.21%)	0.17% (0.12% , 0.25%)
94	39,697	0.24% (0.19% , 0.29%)	0.26% (0.21% , 0.33%)	35	38,793	0.09% (0.06% , 0.13%)	0.09% (0.06% , 0.13%)
119	36,642	0.32% (0.27% , 0.39%)	0.36% (0.29% , 0.44%)	18	31,128	0.06% (0.03% , 0.09%)	0.07% (0.04% , 0.12%)
41	5,836	0.70% (0.52% , 0.95%)	0.69% (0.49% , 0.98%)	16	4,935	0.32% (0.19% , 0.53%)	0.41% (0.24% , 0.70%)
112	19,402	0.58% (0.48% , 0.69%)	0.69% (0.54% , 0.88%)	41	16,106	0.25% (0.18% , 0.35%)	0.31% (0.21% , 0.45%)
42	11,218	0.37% (0.28% , 0.51%)	0.60% (0.37% , 0.96%)	28	9,601	0.29% (0.19% , 0.42%)	0.36% (0.22% , 0.57%)
103	21,193	0.49% (0.40% , 0.59%)	0.59% (0.45% , 0.77%)	34	17,934	0.19% (0.13% , 0.26%)	0.19% (0.13% , 0.28%)
60	15,478	0.39% (0.30% , 0.50%)	0.36% (0.25% , 0.52%)	26	12,926	0.20% (0.13% , 0.29%)	0.20% (0.13% , 0.31%)
86	23,672	0.36% (0.29% , 0.45%)	0.47% (0.36% , 0.60%)	27	20,103	0.13% (0.09% , 0.20%)	0.15% (0.10% , 0.24%)
91	15,492	0.59% (0.48% , 0.72%)	0.60% (0.48% , 0.76%)	22	14,062	0.16% (0.10% , 0.24%)	0.16% (0.10% , 0.26%)
35	16,523	0.21% (0.15% , 0.29%)	0.23% (0.15% , 0.36%)	15	14,049	0.11% (0.06% , 0.18%)	0.13% (0.07% , 0.26%)
55	9,042	0.61% (0.47% , 0.79%)	0.73% (0.49% , 1.08%)	19	8,614	0.22% (0.13% , 0.34%)	0.21% (0.12% , 0.36%)
165	31,393	0.53% (0.45% , 0.61%)	0.57% (0.46% , 0.70%)	44	24,094	0.18% (0.13% , 0.25%)	0.20% (0.14% , 0.30%)
244	59,907	0.41% (0.36% , 0.46%)	0.47% (0.40% , 0.56%)	90	52,495	0.17% (0.14% , 0.21%)	0.23% (0.18% , 0.30%)
202	60,496	0.33% (0.29% , 0.38%)	0.43% (0.36% , 0.52%)	64	52,331	0.12% (0.09% , 0.16%)	0.14% (0.11% , 0.19%)
23	4,618	0.50% (0.33% , 0.75%)	0.55% (0.33% , 0.92%)	10	3,310	0.30% (0.15% , 0.55%)	0.33% (0.17% , 0.64%)
596	149,859	0.40% (0.37% , 0.43%)	0.45% (0.41% , 0.50%)	194	126,966	0.15% (0.13% , 0.18%)	0.19% (0.16% , 0.23%)
52	6,516	0.80% (0.61% , 1.04%)	0.95% (0.64% , 1.41%)	14	5,892	0.24% (0.13% , 0.40%)	0.27% (0.15% , 0.47%)
14	2,151	0.65% (0.39% , 1.09%)	0.79% (0.39% , 1.60%)	4	1,965	0.20% (0.06% , 0.52%)	NA (NA , NA)
8	2,926	0.27% (0.14% , 0.54%)	NA (NA , NA)	7	2,460	0.28% (0.11% , 0.59%)	NA (NA , NA)
5	1,185	0.42% (0.18% , 0.98%)	NA (NA , NA)	3	1,143	0.26% (0.05% , 0.77%)	NA (NA , NA)
14	2,819	0.50% (0.30% , 0.83%)	0.54% (0.30% , 0.94%)	5	2,418	0.21% (0.07% , 0.48%)	NA (NA , NA)
68	23,576	0.29% (0.23% , 0.37%)	0.31% (0.24% , 0.42%)	26	20,336	0.13% (0.08% , 0.19%)	0.15% (0.09% , 0.24%)
177	59,046	0.30% (0.26% , 0.35%)	0.32% (0.27% , 0.38%)	64	53,128	0.12% (0.09% , 0.15%)	0.16% (0.11% , 0.22%)
145	30,652	0.47% (0.40% , 0.56%)	0.52% (0.42% , 0.64%)	48	25,373	0.19% (0.14% , 0.25%)	0.22% (0.15% , 0.33%)
182	35,280	0.52% (0.45% , 0.60%)	0.56% (0.46% , 0.67%)	58	28,568	0.20% (0.15% , 0.26%)	0.24% (0.18% , 0.33%)
83	12,055	0.69% (0.56% , 0.85%)	0.84% (0.62% , 1.12%)	19	9,582	0.20% (0.12% , 0.31%)	0.21% (0.11% , 0.38%)
34	4,847	0.70% (0.50% , 0.98%)	1.41% (0.88% , 2.26%)	12	3,857	0.31% (0.16% , 0.54%)	0.35% (0.19% , 0.65%)
350	139,231	0.25% (0.23% , 0.28%)	0.29% (0.25% , 0.33%)	136	119,963	0.11% (0.10% , 0.13%)	0.14% (0.11% , 0.17%)
215	2,587	8.31% (7.31% , 9.44%)	9.41% (7.85% , 11.23%)	46	977	4.71% (3.47% , 6.23%)	4.86% (3.37% , 6.96%)
15	640	2.34% (1.43% , 3.83%)	3.07% (1.02% , 8.90%)	5	397	1.26% (0.41% , 2.91%)	NA (NA , NA)
109	22,998	0.47% (0.39% , 0.57%)	0.52% (0.41% , 0.66%)	40	19,507	0.21% (0.15% , 0.28%)	0.27% (0.18% , 0.40%)
223	4,968	4.49% (3.95% , 5.10%)	4.89% (4.02% , 5.92%)	45	3,508	1.28% (0.94% , 1.71%)	1.56% (1.09% , 2.24%)
105	17,099	0.61% (0.51% , 0.74%)	0.74% (0.56% , 0.96%)	30	14,801	0.20% (0.14% , 0.29%)	0.28% (0.18% , 0.42%)
256	120,482	0.21% (0.19% , 0.24%)	0.26% (0.22% , 0.31%)	112	103,135	0.11% (0.09% , 0.13%)	0.12% (0.10% , 0.15%)
105	22,907	0.46% (0.38% , 0.55%)	0.50% (0.40% , 0.64%)	40	19,400	0.21% (0.15% , 0.28%)	0.27% (0.18% , 0.40%)
108	15,718	0.69% (0.57% , 0.83%)	0.85% (0.66% , 1.09%)	36	12,974	0.28% (0.19% , 0.38%)	0.33% (0.23% , 0.47%)
142	25,369	0.56% (0.48% , 0.66%)	0.59% (0.49% , 0.72%)	46	21,750	0.21% (0.15% , 0.28%)	0.25% (0.18% , 0.35%)
122	35,741	0.34% (0.29% , 0.41%)	0.34% (0.28% , 0.42%)	44	30,461	0.14% (0.11% , 0.19%)	0.17% (0.12% , 0.24%)
157	41,455	0.38% (0.32% , 0.44%)	0.38% (0.32% , 0.46%)	48	35,151	0.14% (0.10% , 0.18%)	0.13% (0.09% , 0.17%)
160	47,173	0.34% (0.29% , 0.40%)	0.36% (0.30% , 0.43%)	53	40,508	0.13% (0.10% , 0.17%)	0.13% (0.09% , 0.17%)
