# Supplementary material for: Health inequalities in risk of SARS-CoV-2 infection in England’s second wave: population-based cross-sectional analysis from the REACT-1 study
Source: BMJ Public Health. 2026 Jan 5;4(1):e002596. doi: 10.1136/bmjph-2025-002596 (PMC13663695; doi:10.1136/bmjph-2025-002596)
Supplement: Supplementary file 4 [file bmjph-4-1-s004.pdf]

Supplementary Table 3. Odds ratios for rounds 5 to 10, and an average weighted inversely to variance for core variables.

Variable	Category	Round 5		Round 6		Round 7		Round
		Adjusted for age and sex	Mutually adjusted	Adjusted for age and sex	Mutually adjusted	Adjusted for age and sex	Mutually adjusted	Adjusted for age and sex
Sex	Male	Ref	Ref	Ref	Ref	Ref	Ref	Ref
	Female	0.90 [0.78,1.03]	0.90 [0.78,1.03]	1.01 [0.92,1.11]	1.01 [0.92,1.12]	0.95 [0.85,1.06]	0.94 [0.84,1.05]	0.94 [0.87,1.02]
Age Group	5-12	0.80 [0.59,1.10]	0.75 [0.54,1.02]	0.99 [0.78,1.24]	0.95 [0.75,1.19]	1.61 [1.29,2.02]	1.50 [1.20,1.89]	1.00 [0.83,1.21]
	13-17	1.06 [0.77,1.45]	0.97 [0.69,1.37]	1.36 [1.09,1.71]	1.38 [1.07,1.77]	2.29 [1.84,2.84]	2.30 [1.79,2.95]	1.33 [1.11,1.61]
	18-24	2.31 [1.75,3.06]	2.17 [1.63,2.88]	1.88 [1.50,2.36]	1.87 [1.49,2.35]	1.74 [1.34,2.27]	1.74 [1.34,2.27]	1.62 [1.33,1.96]
	25-34	1.04 [0.79,1.38]	1.10 [0.83,1.45]	1.33 [1.10,1.61]	1.37 [1.12,1.66]	1.15 [0.91,1.45]	1.17 [0.93,1.48]	1.22 [1.03,1.44]
	35-44	Ref	Ref	Ref	Ref	Ref	Ref	Ref
	45-54	1.01 [0.80,1.28]	1.07 [0.84,1.36]	1.14 [0.96,1.36]	1.16 [0.97,1.38]	1.05 [0.85,1.28]	1.10 [0.90,1.36]	1.02 [0.88,1.18]
	55-64	0.69 [0.53,0.88]	0.78 [0.60,1.02]	1.08 [0.91,1.28]	1.17 [0.97,1.40]	0.78 [0.63,0.96]	0.93 [0.74,1.16]	0.89 [0.77,1.03]
	65+	0.61 [0.48,0.78]	0.75 [0.56,1.00]	0.76 [0.64,0.91]	0.92 [0.75,1.13]	0.56 [0.45,0.68]	0.79 [0.62,1.01]	0.60 [0.52,0.70]
Region	North East	3.46 [2.52,4.76]	3.48 [2.52,4.81]	2.28 [1.79,2.91]	2.19 [1.71,2.80]	1.47 [1.09,1.97]	1.30 [0.96,1.76]	0.74 [0.57,0.96]
	North West	3.92 [3.09,4.98]	3.94 [3.09,5.02]	3.15 [2.67,3.70]	3.03 [2.56,3.57]	1.34 [1.10,1.64]	1.21 [0.99,1.49]	0.84 [0.72,0.98]
	Yorkshire and The Humber	2.44 [1.81,3.29]	2.52 [1.86,3.40]	2.67 [2.20,3.23]	2.62 [2.16,3.18]	1.47 [1.17,1.85]	1.40 [1.11,1.76]	0.60 [0.48,0.74]
	East Midlands	1.76 [1.34,2.32]	1.79 [1.36,2.36]	1.70 [1.42,2.03]	1.68 [1.40,2.02]	1.54 [1.28,1.84]	1.48 [1.24,1.78]	0.71 [0.60,0.82]
	West Midlands	1.62 [1.20,2.20]	1.62 [1.20,2.20]	1.97 [1.63,2.38]	1.92 [1.59,2.32]	1.52 [1.24,1.86]	1.43 [1.17,1.75]	0.89 [0.76,1.05]
	East of England	1.19 [0.89,1.60]	1.22 [0.91,1.63]	0.95 [0.78,1.17]	0.94 [0.77,1.16]	0.76 [0.61,0.95]	0.75 [0.60,0.93]	1.05 [0.92,1.20]
	London	1.63 [1.21,2.18]	1.47 [1.09,1.99]	1.26 [1.03,1.56]	1.20 [0.96,1.48]	1.24 [1.01,1.53]	1.09 [0.87,1.35]	1.62 [1.42,1.84]
	South East	Ref	Ref	Ref	Ref	Ref	Ref	Ref
	South West	1.32 [0.95,1.83]	1.40 [1.00,1.94]	1.15 [0.92,1.43]	1.14 [0.92,1.43]	0.84 [0.66,1.07]	0.82 [0.64,1.05]	0.52 [0.42,0.63]
Key Worker Status	HCW/CHW*	1.15 [0.85,1.55]	1.03 [0.76,1.38]	1.27 [1.04,1.54]	1.16 [0.95,1.41]	1.74 [1.42,2.14]	1.66 [1.35,2.03]	1.47 [1.24,1.74]
	Other essential/key workers	1.09 [0.90,1.32]	1.03 [0.85,1.25]	1.24 [1.09,1.42]	1.16 [1.01,1.32]	1.19 [1.02,1.39]	1.17 [1.00,1.36]	1.28 [1.15,1.43]
	Other worker	Ref	Ref	Ref	Ref	Ref	Ref	Ref
	Not FT, PT, SE*	1.09 [0.91,1.32]	1.03 [0.86,1.24]	0.97 [0.85,1.10]	0.93 [0.82,1.06]	0.94 [0.80,1.10]	0.91 [0.78,1.06]	0.91 [0.81,1.02]
	Unknown	1.07 [0.69,1.65]	0.97 [0.63,1.50]	1.18 [0.88,1.59]	1.10 [0.81,1.48]	1.48 [1.12,1.95]	1.38 [1.04,1.82]	0.76 [0.57,1.03]
Ethnicity	White	Ref	Ref	Ref	Ref	Ref	Ref	Ref
	Asian	1.81 [1.41,2.31]	1.89 [1.45,2.45]	1.10 [0.88,1.37]	1.19 [0.95,1.50]	1.67 [1.35,2.07]	1.53 [1.23,1.91]	1.79 [1.52,2.10]
	Black	1.51 [0.92,2.49]	1.58 [0.95,2.63]	1.16 [0.77,1.76]	1.24 [0.81,1.89]	1.20 [0.75,1.91]	1.02 [0.63,1.65]	2.40 [1.85,3.12]
	Mixed	0.70 [0.38,1.27]	0.79 [0.43,1.44]	0.88 [0.60,1.29]	0.98 [0.67,1.43]	0.82 [0.54,1.25]	0.84 [0.55,1.28]	1.14 [0.85,1.53]
	Other	1.03 [0.49,2.17]	1.08 [0.51,2.30]	1.31 [0.81,2.11]	1.45 [0.89,2.36]	1.72 [1.04,2.83]	1.66 [1.00,2.74]	1.80 [1.24,2.61]
	Unknown	1.09 [0.61,1.92]	1.11 [0.63,1.97]	1.20 [0.83,1.73]	1.24 [0.86,1.79]	1.49 [1.00,2.22]	1.44 [0.96,2.15]	1.20 [0.89,1.61]
Household Size	1-2 People	Ref	Ref	Ref	Ref	Ref	Ref	Ref
	3-5 People	1.28 [1.08,1.53]	1.30 [1.10,1.55]	1.14 [1.01,1.28]	1.18 [1.05,1.32]	1.24 [1.08,1.43]	1.26 [1.09,1.46]	1.41 [1.27,1.56]
	6+ People	2.01 [1.46,2.78]	1.84 [1.32,2.56]	1.44 [1.11,1.86]	1.47 [1.13,1.90]	2.07 [1.60,2.67]	1.91 [1.47,2.47]	2.17 [1.76,2.67]
Deprivation Index Quintile	1 - Most Deprived	1.60 [1.29,1.98]	1.11 [0.89,1.39]	1.66 [1.42,1.95]	1.25 [1.07,1.47]	1.89 [1.58,2.27]	1.69 [1.40,2.04]	1.41 [1.22,1.64]
	2	1.00 [0.81,1.25]	0.86 [0.69,1.08]	1.20 [1.03,1.39]	1.08 [0.93,1.26]	1.41 [1.19,1.68]	1.35 [1.13,1.60]	1.47 [1.29,1.66]
	3	0.85 [0.69,1.05]	0.81 [0.65,0.99]	1.08 [0.94,1.25]	1.04 [0.90,1.20]	1.19 [1.01,1.40]	1.17 [0.99,1.39]	1.15 [1.01,1.30]
	4	0.97 [0.80,1.18]	0.91 [0.75,1.11]	1.08 [0.95,1.24]	1.02 [0.89,1.17]	1.11 [0.94,1.30]	1.09 [0.93,1.28]	1.12 [0.99,1.26]
	5 - Least Deprived	Ref	Ref	Ref	Ref	Ref	Ref	Ref

* We present weighted prevalence if the number of positives in a category is 10 or more.

8	Round 9		Round 10		Rounds 5 to 10	
Mutually adjusted	Adjusted for age and sex	Mutually adjusted	Adjusted for age and sex	Mutually adjusted	Adjusted for age and sex	Mutually adjusted
Ref	Ref	Ref	Ref	Ref	Ref	Ref
0.94 [0.86,1.02]	0.97 [0.83,1.12]	0.97 [0.83,1.13]	0.87 [0.67,1.14]	0.88 [0.68,1.15]	0.95 [0.91, 1.00]	0.95 [0.91, 1.00]
0.92 [0.76,1.11]	1.33 [0.99,1.80]	1.24 [0.91,1.68]	1.50 [0.93,2.42]	1.38 [0.85,2.25]	1.15 [0.93, 1.43]	1.07 [0.87, 1.33]
1.55 [1.26,1.91]	0.99 [0.70,1.42]	1.01 [0.68,1.49]	0.87 [0.47,1.63]	0.74 [0.37,1.48]	1.3 [0.99, 1.71]	1.31 [0.96, 1.77]
1.66 [1.36,2.02]	1.33 [0.91,1.92]	1.35 [0.93,1.96]	1.11 [0.59,2.11]	1.06 [0.56,2.02]	1.73 [1.49, 2.01]	1.74 [1.55, 1.94]
1.26 [1.07,1.49]	1.03 [0.75,1.41]	1.08 [0.79,1.48]	0.86 [0.50,1.47]	0.86 [0.50,1.48]	1.18 [1.07, 1.30]	1.22 [1.10, 1.34]
Ref	Ref	Ref	Ref	Ref	Ref	Ref
1.11 [0.96,1.29]	1.07 [0.82,1.40]	1.16 [0.89,1.52]	0.82 [0.52,1.30]	0.85 [0.54,1.36]	1.05 [0.96, 1.14]	1.11 [1.02, 1.21]
1.16 [0.99,1.35]	0.95 [0.73,1.24]	1.24 [0.94,1.63]	0.74 [0.47,1.17]	0.84 [0.52,1.35]	0.87 [0.75, 1.00]	1.03 [0.89, 1.2]
0.98 [0.82,1.17]	0.53 [0.40,0.70]	0.85 [0.61,1.19]	0.44 [0.28,0.71]	0.55 [0.31,0.96]	0.6 [0.53, 0.69]	0.86 [0.77, 0.97]
0.69 [0.53,0.90]	2.20 [1.54,3.14]	1.92 [1.34,2.76]	5.75 [2.93,11.28]	5.32 [2.68,10.53]	2.11 [1.22, 3.65]	1.97 [1.13, 3.44]
0.79 [0.67,0.92]	1.81 [1.40,2.34]	1.62 [1.24,2.11]	4.51 [2.59,7.85]	4.20 [2.39,7.37]	2.16 [1.28, 3.65]	2.03 [1.17, 3.5]
0.58 [0.46,0.72]	1.17 [0.82,1.67]	1.11 [0.78,1.58]	5.16 [2.85,9.33]	5.03 [2.77,9.14]	1.77 [0.98, 3.17]	1.72 [0.95, 3.13]
0.69 [0.59,0.80]	1.51 [1.16,1.97]	1.46 [1.12,1.90]	3.33 [1.88,5.89]	3.29 [1.85,5.84]	1.53 [1.06, 2.23]	1.51 [1.03, 2.21]
0.86 [0.73,1.01]	1.20 [0.88,1.64]	1.12 [0.82,1.53]	3.53 [1.93,6.44]	3.41 [1.86,6.24]	1.55 [1.12, 2.16]	1.49 [1.06, 2.1]
1.03 [0.90,1.17]	1.12 [0.85,1.48]	1.10 [0.83,1.45]	2.35 [1.29,4.27]	2.31 [1.27,4.19]	1.06 [0.87, 1.3]	1.05 [0.86, 1.3]
1.45 [1.26,1.66]	1.73 [1.32,2.28]	1.54 [1.16,2.05]	2.52 [1.35,4.70]	2.18 [1.15,4.13]	1.51 [1.31, 1.73]	1.35 [1.18, 1.54]
Ref	Ref	Ref	Ref	Ref	Ref	Ref
0.50 [0.42,0.61]	0.66 [0.46,0.97]	0.65 [0.44,0.95]	1.90 [0.95,3.76]	1.90 [0.95,3.77]	0.93 [0.65, 1.32]	0.93 [0.64, 1.34]
1.48 [1.25,1.76]	1.42 [1.06,1.92]	1.36 [1.01,1.83]	1.21 [0.73,1.99]	1.09 [0.66,1.81]	1.41 [1.24, 1.6]	1.32 [1.13, 1.54]
1.35 [1.21,1.51]	1.22 [1.00,1.49]	1.18 [0.97,1.44]	0.98 [0.68,1.41]	0.89 [0.62,1.29]	1.22 [1.14, 1.3]	1.17 [1.07, 1.29]
Ref	Ref	Ref	Ref	Ref	Ref	Ref
0.93 [0.83,1.04]	0.99 [0.80,1.22]	0.96 [0.78,1.18]	0.95 [0.66,1.36]	0.89 [0.62,1.27]	0.96 [0.9, 1.02]	0.94 [0.88, 1.00]
0.76 [0.56,1.02]	1.11 [0.71,1.73]	1.03 [0.66,1.62]	1.79 [0.88,3.64]	1.61 [0.79,3.27]	1.14 [0.91, 1.44]	1.07 [0.86, 1.32]
Ref	Ref	Ref	Ref	Ref	Ref	Ref
1.36 [1.15,1.61]	1.81 [1.36,2.42]	1.53 [1.14,2.07]	1.31 [0.76,2.27]	1.29 [0.73,2.27]	1.58 [1.32, 1.89]	1.45 [1.27, 1.66]
1.61 [1.23,2.10]	1.48 [0.87,2.52]	1.10 [0.64,1.90]	1.13 [0.42,3.06]	1.03 [0.37,2.83]	1.52 [1.13, 2.03]	1.34 [1.11, 1.63]
0.99 [0.74,1.34]	0.58 [0.29,1.16]	0.56 [0.28,1.13]	1.41 [0.66,3.03]	1.57 [0.73,3.38]	0.92 [0.75, 1.13]	0.93 [0.77, 1.11]
1.35 [0.93,1.97]	0.97 [0.40,2.36]	0.83 [0.34,2.01]	1.52 [0.49,4.78]	1.53 [0.48,4.87]	1.50 [1.20, 1.89]	1.36 [1.08, 1.72]
1.10 [0.82,1.48]	1.34 [0.79,2.28]	1.26 [0.74,2.15]	1.44 [0.59,3.52]	1.43 [0.59,3.48]	1.26 [1.06, 1.5]	1.22 [1.03, 1.45]
Ref	Ref	Ref	Ref	Ref	Ref	Ref
1.40 [1.26,1.55]	1.55 [1.29,1.88]	1.59 [1.31,1.92]	1.16 [0.83,1.61]	1.22 [0.88,1.70]	1.30 [1.18, 1.43]	1.32 [1.21, 1.44]
1.95 [1.58,2.41]	2.08 [1.42,3.05]	1.93 [1.31,2.84]	1.72 [0.90,3.28]	1.68 [0.87,3.24]	1.92 [1.64, 2.24]	1.80 [1.60, 2.03]
1.44 [1.24,1.68]	1.95 [1.52,2.49]	1.72 [1.33,2.21]	2.00 [1.31,3.06]	1.44 [0.93,2.24]	1.69 [1.51, 1.89]	1.42 [1.23, 1.63]
1.41 [1.24,1.61]	1.62 [1.29,2.03]	1.52 [1.21,1.92]	1.57 [1.05,2.33]	1.37 [0.91,2.04]	1.34 [1.17, 1.54]	1.24 [1.04, 1.47]
1.15 [1.02,1.31]	1.01 [0.79,1.27]	1.00 [0.79,1.27]	1.10 [0.74,1.64]	1.01 [0.68,1.51]	1.07 [0.98, 1.18]	1.04 [0.93, 1.17]
1.14 [1.01,1.29]	1.12 [0.90,1.40]	1.11 [0.89,1.38]	1.05 [0.71,1.56]	0.97 [0.65,1.43]	1.09 [1.02, 1.16]	1.06 [0.99, 1.14]
Ref	Ref	Ref	Ref	Ref	Ref	Ref
