# Supplementary material for: Health inequalities in risk of SARS-CoV-2 infection in England’s second wave: population-based cross-sectional analysis from the REACT-1 study
Source: BMJ Public Health. 2026 Jan 5;4(1):e002596. doi: 10.1136/bmjph-2025-002596 (PMC13663695; doi:10.1136/bmjph-2025-002596)
Supplement: Supplementary file 5 [file bmjph-4-1-s005.pdf]

Supplementary Table 4. The unweighted and weighted prevalence of swab-positivity for detailed ethnicity subgroups for rounds 5 to 10.

Variable	Category	Round 5				Round 6				Round 7			
		Positive	Total	Unweighted Prevalence	Weighted Prevalence*	Positive	Total	Unweighted Prevalence	Weighted Prevalence*	Positive	Total	Unweighted Prevalence	Weighted Prevalence*
Ethnic group	White	702	157,370	0.45% (0.41% , 0.48%)	0.54% (0.48% , 0.59%)	1550	145,336	1.07% (1.02% , 1.12%)	1.24% (1.16% , 1.33%)	1122	153,463	0.73% (0.69% , 0.78%)	0.84% (0.78% , 0.90%)
	Asian	72	7,891	0.91% (0.73% , 1.15%)	1.19% (0.89% , 1.60%)	84	6,712	1.25% (1.01% , 1.55%)	2.05% (1.45% , 2.88%)	95	6,577	1.44% (1.18% , 1.76%)	2.07% (1.57% , 2.71%)
	Black	16	2,187	0.73% (0.45% , 1.19%)	1.17% (0.65% , 2.11%)	23	1,760	1.31% (0.87% , 1.95%)	1.23% (0.76% , 1.98%)	18	1,754	1.03% (0.65% , 1.62%)	1.07% (0.65% , 1.76%)
	Mixed	11	3,071	0.36% (0.20% , 0.64%)	0.44% (0.23% , 0.87%)	28	2,697	1.04% (0.72% , 1.50%)	1.01% (0.66% , 1.53%)	23	2,721	0.85% (0.56% , 1.27%)	0.84% (0.53% , 1.33%)
	Other	6	1,435	0.42% (0.19% , 0.91%)	0.41% (0.17% , 0.98%)	17	1,184	1.44% (0.90% , 2.29%)	1.81% (1.00% , 3.24%)	16	1,157	1.38% (0.85% , 2.23%)	1.97% (1.09% , 3.51%)
	NA	11	2,676	0.41% (0.23% , 0.73%)	0.62% (0.25% , 1.53%)	30	2,486	1.21% (0.85% , 1.72%)	1.38% (0.87% , 2.18%)	25	2,509	1.00% (0.68% , 1.47%)	1.15% (0.74% , 1.78%)
White ethnic group	English/Welsh/Scottish/ Northern Irish/British	663	147,483	0.45% (0.42% , 0.49%)	0.53% (0.48% , 0.59%)	1445	136,000	1.06% (1.01% , 1.12%)	1.24% (1.15% , 1.33%)	1052	144,148	0.73% (0.69% , 0.78%)	0.83% (0.77% , 0.90%)
	Irish	7	1,672	0.42% (0.17% , 0.86%)	NA (NA , NA)	16	1,458	1.10% (0.63% , 1.78%)	1.23% (0.71% , 2.13%)	5	1,600	0.31% (0.10% , 0.73%)	NA (NA , NA)
	Gypsy or Irish Traveller	0	48	0.00% (0.00% , 7.40%)	NA (NA , NA)	0	45	0.00% (0.00% , 7.87%)	NA (NA , NA)	1	49	2.04% (0.05% , 10.85%)	NA (NA , NA)
	Other white background	32	8,167	0.39% (0.27% , 0.55%)	0.59% (0.38% , 0.94%)	88	7,831	1.12% (0.90% , 1.38%)	1.37% (1.07% , 1.75%)	64	7,666	0.83% (0.64% , 1.06%)	0.96% (0.72% , 1.28%)
Asian ethnic group	Indian	26	3,849	0.68% (0.44% , 0.99%)	0.75% (0.49% , 1.13%)	34	3,304	1.03% (0.71% , 1.44%)	1.34% (0.88% , 2.03%)	42	3,250	1.29% (0.93% , 1.74%)	1.35% (0.95% , 1.91%)
	Pakistani	16	1,013	1.58% (0.91% , 2.55%)	1.94% (1.07% , 3.51%)	20	792	2.53% (1.55% , 3.87%)	3.42% (1.81% , 6.34%)	17	706	2.41% (1.41% , 3.83%)	2.52% (1.47% , 4.28%)
	Bangladeshi	14	499	2.81% (1.54% , 4.66%)	2.26% (1.24% , 4.08%)	6	330	1.82% (0.67% , 3.92%)	NA (NA , NA)	16	355	4.51% (2.60% , 7.22%)	4.56% (2.67% , 7.69%)
	Chinese	3	867	0.35% (0.07% , 1.01%)	NA (NA , NA)	5	884	0.57% (0.18% , 1.32%)	NA (NA , NA)	3	807	0.37% (0.08% , 1.08%)	NA (NA , NA)
	Other Asian background	13	1,663	0.78% (0.42% , 1.33%)	0.89% (0.49% , 1.61%)	19	1,402	1.36% (0.82% , 2.11%)	1.36% (0.76% , 2.44%)	17	1,459	1.17% (0.68% , 1.86%)	2.34% (1.06% , 5.07%)
Black ethnic group	African	13	1,417	0.92% (0.49% , 1.56%)	1.54% (0.80% , 2.94%)	17	1,145	1.48% (0.87% , 2.37%)	1.13% (0.65% , 1.96%)	16	1,082	1.48% (0.85% , 2.39%)	1.45% (0.85% , 2.46%)
	Caribbean	3	585	0.51% (0.11% , 1.49%)	NA (NA , NA)	6	465	1.29% (0.48% , 2.79%)	NA (NA , NA)	2	509	0.39% (0.05% , 1.41%)	NA (NA , NA)
	Other Black/African/Caribbean background	0	185	0.00% (0.00% , 1.97%)	NA (NA , NA)	0	150	0.00% (0.00% , 2.43%)	NA (NA , NA)	0	163	0.00% (0.00% , 2.24%)	NA (NA , NA)
Mixed ethinic group	White and Black Caribbean	2	639	0.31% (0.04% , 1.13%)	NA (NA , NA)	9	532	1.69% (0.78% , 3.19%)	NA (NA , NA)	5	541	0.92% (0.30% , 2.14%)	NA (NA , NA)
	White and Black African	0	316	0.00% (0.00% , 1.16%)	NA (NA , NA)	2	278	0.72% (0.09% , 2.57%)	NA (NA , NA)	1	303	0.33% (0.01% , 1.83%)	NA (NA , NA)
	White and Asian	3	1,095	0.27% (0.06% , 0.80%)	NA (NA , NA)	9	951	0.95% (0.43% , 1.79%)	NA (NA , NA)	11	973	1.13% (0.57% , 2.01%)	1.36% (0.71% , 2.59%)
	Other Mixed/Multiple ethnic background	6	1,021	0.59% (0.22% , 1.27%)	NA (NA , NA)	8	936	0.85% (0.37% , 1.68%)	NA (NA , NA)	6	904	0.66% (0.24% , 1.44%)	NA (NA , NA)
Other	Arab	4	349	1.15% (0.31% , 2.91%)	NA (NA , NA)	3	299	1.00% (0.21% , 2.90%)	NA (NA , NA)	4	278	1.44% (0.39% , 3.64%)	NA (NA , NA)
	Other ethnic group	2	1,086	0.18% (0.02% , 0.66%)	NA (NA , NA)	14	937	1.49% (0.82% , 2.49%)	1.84% (0.94% , 3.58%)	12	940	1.28% (0.66% , 2.22%)	1.87% (0.94% , 3.66%)
	Prefer not to say	4	1,314	0.30% (0.08% , 0.78%)	NA (NA , NA)	17	1,164	1.46% (0.85% , 2.33%)	1.66% (0.91% , 3.01%)	15	1,075	1.40% (0.78% , 2.29%)	1.36% (0.75% , 2.43%)
	NA	7	1,362	0.51% (0.21% , 1.06%)	NA (NA , NA)	13	1,270	1.02% (0.55% , 1.74%)	1.15% (0.55% , 2.36%)	10	1,373	0.73% (0.35% , 1.34%)	1.02% (0.52% , 1.99%)

* We present weighted prevalence if the number of positives in a category is 10 or more.

Round 8			
Positive	Total	Unweighted Prevalence	Weighted Prevalence*
1938	152,111	1.27% (1.22% , 1.33%)	1.41% (1.33% , 1.49%)
163	6,537	2.49% (2.14% , 2.90%)	2.80% (2.32% , 3.37%)
60	1,832	3.28% (2.55% , 4.19%)	3.07% (2.29% , 4.09%)
46	2,751	1.67% (1.26% , 2.22%)	1.78% (1.28% , 2.46%)
29	1,190	2.44% (1.70% , 3.48%)	2.42% (1.59% , 3.65%)
46	3,221	1.43% (1.07% , 1.90%)	1.76% (1.23% , 2.50%)
1752	142,570	1.23% (1.17% , 1.29%)	1.35% (1.27% , 1.44%)
30	1,558	1.93% (1.30% , 2.74%)	2.15% (1.40% , 3.29%)
0	42	0.00% (0.00% , 8.41%)	NA (NA , NA)
156	7,941	1.96% (1.67% , 2.29%)	2.14% (1.78% , 2.56%)
69	3,183	2.17% (1.69% , 2.74%)	2.42% (1.82% , 3.21%)
19	728	2.61% (1.58% , 4.05%)	2.29% (1.40% , 3.72%)
20	327	6.12% (3.78% , 9.29%)	6.03% (3.77% , 9.52%)
9	811	1.11% (0.51% , 2.10%)	NA (NA , NA)
46	1,488	3.09% (2.27% , 4.10%)	3.02% (2.16% , 4.20%)
42	1,123	3.74% (2.71% , 5.02%)	3.43% (2.41% , 4.86%)
12	553	2.17% (1.13% , 3.76%)	1.90% (1.02% , 3.52%)
6	156	3.85% (1.42% , 8.18%)	NA (NA , NA)
7	489	1.43% (0.58% , 2.93%)	NA (NA , NA)
5	292	1.71% (0.56% , 3.95%)	NA (NA , NA)
18	1,000	1.80% (1.07% , 2.83%)	1.90% (1.11% , 3.23%)
16	970	1.65% (0.95% , 2.66%)	1.98% (1.15% , 3.39%)
6	296	2.03% (0.75% , 4.36%)	NA (NA , NA)
23	930	2.47% (1.57% , 3.69%)	3.02% (2.16% , 4.20%)
28	1,430	1.96% (1.31% , 2.82%)	2.55% (1.64% , 3.96%)
18	1,755	1.03% (0.61% , 1.62%)	0.99% (0.57% , 1.70%)

Round 9			
Positive	Total	Unweighted Prevalence	Weighted Prevalence*
596	149,859	0.40% (0.37% , 0.43%)	0.45% (0.40% , 0.50%)
52	6,516	0.80% (0.61% , 1.04%)	0.91% (0.61% , 1.36%)
14	2,151	0.65% (0.39% , 1.09%)	0.83% (0.41% , 1.68%)
8	2,926	0.27% (0.14% , 0.54%)	NA (NA , NA)
5	1,185	0.42% (0.18% , 0.98%)	NA (NA , NA)
14	2,819	0.50% (0.30% , 0.83%)	0.51% (0.28% , 0.92%)
524	140,193	0.37% (0.34% , 0.41%)	0.41% (0.37% , 0.46%)
6	1,428	0.42% (0.15% , 0.91%)	NA (NA , NA)
0	37	0.00% (0.00% , 9.49%)	NA (NA , NA)
66	8,201	0.80% (0.62% , 1.02%)	0.95% (0.71% , 1.27%)
26	3,119	0.83% (0.55% , 1.22%)	0.72% (0.47% , 1.10%)
11	818	1.34% (0.67% , 2.39%)	2.08% (1.02% , 4.20%)
3	351	0.85% (0.18% , 2.48%)	NA (NA , NA)
1	748	0.13% (0.00% , 0.74%)	NA (NA , NA)
11	1,480	0.74% (0.37% , 1.33%)	0.55% (0.29% , 1.04%)
11	1,417	0.78% (0.39% , 1.38%)	1.02% (0.47% , 2.21%)
1	588	0.17% (0.00% , 0.94%)	NA (NA , NA)
2	146	1.37% (0.17% , 4.86%)	NA (NA , NA)
1	540	0.19% (0.00% , 1.03%)	NA (NA , NA)
0	323	0.00% (0.00% , 1.14%)	NA (NA , NA)
5	1,072	0.47% (0.15% , 1.09%)	NA (NA , NA)
2	991	0.20% (0.02% , 0.73%)	NA (NA , NA)
4	314	1.27% (0.35% , 3.23%)	NA (NA , NA)
2	916	0.22% (0.03% , 0.79%)	NA (NA , NA)
7	1,125	0.62% (0.25% , 1.28%)	NA (NA , NA)
6	1,649	0.36% (0.13% , 0.79%)	NA (NA , NA)

Round 10			
Positive	Total	Unweighted Prevalence	Weighted Prevalence*
194	126,966	0.15% (0.13% , 0.18%)	0.19% (0.16% , 0.23%)
14	5,892	0.24% (0.14% , 0.40%)	0.27% (0.15% , 0.47%)
4	1,965	0.20% (0.08% , 0.52%)	NA (NA , NA)
7	2,460	0.28% (0.14% , 0.59%)	NA (NA , NA)
3	1,143	0.26% (0.09% , 0.77%)	NA (NA , NA)
5	2,418	0.21% (0.09% , 0.48%)	NA (NA , NA)
179	118,326	0.15% (0.13% , 0.18%)	0.19% (0.16% , 0.23%)
1	1,376	0.07% (0.00% , 0.40%)	NA (NA , NA)
0	24	0.00% (0.00% , 14.25%)	NA (NA , NA)
14	7,240	0.19% (0.11% , 0.32%)	0.27% (0.14% , 0.51%)
5	2,788	0.18% (0.06% , 0.42%)	NA (NA , NA)
3	749	0.40% (0.08% , 1.17%)	NA (NA , NA)
2	337	0.59% (0.07% , 2.13%)	NA (NA , NA)
1	718	0.14% (0.00% , 0.77%)	NA (NA , NA)
3	1,300	0.23% (0.05% , 0.67%)	NA (NA , NA)
4	1,282	0.31% (0.09% , 0.80%)	NA (NA , NA)
0	547	0.00% (0.00% , 0.67%)	NA (NA , NA)
0	136	0.00% (0.00% , 2.68%)	NA (NA , NA)
2	479	0.42% (0.05% , 1.50%)	NA (NA , NA)
1	294	0.34% (0.01% , 1.88%)	NA (NA , NA)
2	906	0.22% (0.03% , 0.80%)	NA (NA , NA)
2	781	0.26% (0.03% , 0.92%)	NA (NA , NA)
1	285	0.35% (0.01% , 1.94%)	NA (NA , NA)
2	899	0.22% (0.03% , 0.80%)	NA (NA , NA)
1	899	0.11% (0.00% , 0.62%)	NA (NA , NA)
4	1,478	0.27% (0.07% , 0.69%)	NA (NA , NA)
