# Supplementary material for: Health inequalities in risk of SARS-CoV-2 infection in England’s second wave: population-based cross-sectional analysis from the REACT-1 study
Source: BMJ Public Health. 2026 Jan 5;4(1):e002596. doi: 10.1136/bmjph-2025-002596 (PMC13663695; doi:10.1136/bmjph-2025-002596)
Supplement: Supplementary file 6 [file bmjph-4-1-s006.pdf]

Supplementary Table 5. Odds ratios for rounds 5 to 10, and an average weighted inversely to variance for detailed subgroups of ethnicity.

Variable	Category	Round 5		Round 6		Round 7	
		Adjusted for age and sex	Mutually adjusted	Adjusted for age and sex	Mutually adjusted	Adjusted for age and sex	Mutually adjusted
Gender	Male	Ref	Ref	Ref	Ref	Ref	Ref
	Female	0.90 [0.78,1.03]	0.90 [0.78,1.03]	1.01 [0.92,1.11]	1.01 [0.91,1.11]	0.95 [0.85,1.06]	0.93 [0.83,1.04]
Age Group	5-12	0.80 [0.59,1.10]	0.81 [0.59,1.10]	0.99 [0.78,1.24]	1.00 [0.79,1.25]	1.61 [1.29,2.02]	1.62 [1.29,2.03]
	13-17	1.06 [0.77,1.45]	1.04 [0.74,1.48]	1.36 [1.09,1.71]	1.44 [1.13,1.85]	2.29 [1.84,2.84]	2.48 [1.93,3.18]
	18-24	2.31 [1.75,3.06]	2.25 [1.70,2.99]	1.88 [1.50,2.36]	1.91 [1.52,2.40]	1.74 [1.34,2.27]	1.81 [1.39,2.36]
	25-34	1.04 [0.79,1.38]	1.05 [0.79,1.38]	1.33 [1.10,1.61]	1.33 [1.09,1.61]	1.15 [0.91,1.45]	1.13 [0.89,1.42]
	35-44	Ref	Ref	Ref	Ref	Ref	Ref
	45-54	1.01 [0.80,1.28]	1.03 [0.81,1.31]	1.14 [0.96,1.36]	1.13 [0.95,1.35]	1.05 [0.85,1.28]	1.07 [0.87,1.31]
	55-64	0.69 [0.53,0.88]	0.69 [0.54,0.90]	1.08 [0.91,1.28]	1.09 [0.91,1.29]	0.78 [0.63,0.96]	0.83 [0.67,1.03]
	65+	0.61 [0.48,0.78]	0.63 [0.48,0.82]	0.76 [0.64,0.91]	0.83 [0.68,1.00]	0.56 [0.45,0.68]	0.67 [0.54,0.85]
Region	North East	3.46 [2.52,4.76]	3.44 [2.49,4.75]	2.28 [1.79,2.91]	2.17 [1.69,2.78]	1.47 [1.09,1.97]	1.28 [0.95,1.73]
	North West	3.92 [3.09,4.98]	3.92 [3.07,5.00]	3.15 [2.67,3.70]	3.01 [2.55,3.55]	1.34 [1.10,1.64]	1.20 [0.98,1.47]
	Yorkshire	2.44 [1.81,3.29]	2.49 [1.85,3.37]	2.67 [2.20,3.23]	2.60 [2.14,3.15]	1.47 [1.17,1.85]	1.38 [1.10,1.74]
	East Midlands	1.76 [1.34,2.32]	1.78 [1.35,2.35]	1.70 [1.42,2.03]	1.67 [1.40,2.00]	1.54 [1.28,1.84]	1.48 [1.23,1.77]
	West Midlands	1.62 [1.20,2.20]	1.62 [1.19,2.20]	1.97 [1.63,2.38]	1.92 [1.59,2.32]	1.52 [1.24,1.86]	1.42 [1.16,1.74]
	East of England	1.19 [0.89,1.60]	1.22 [0.91,1.63]	0.95 [0.78,1.17]	0.94 [0.77,1.16]	0.76 [0.61,0.95]	0.75 [0.60,0.92]
	London	1.63 [1.21,2.18]	1.46 [1.08,1.98]	1.26 [1.03,1.56]	1.19 [0.96,1.47]	1.24 [1.01,1.53]	1.07 [0.86,1.34]
	South East	Ref	Ref	Ref	Ref	Ref	Ref
	South West	1.32 [0.95,1.83]	1.39 [1.00,1.93]	1.15 [0.92,1.43]	1.14 [0.91,1.42]	0.84 [0.66,1.07]	0.82 [0.64,1.04]
Key Worker Status	HCW/CHW*	1.15 [0.85,1.55]	1.02 [0.76,1.38]	1.27 [1.04,1.54]	1.16 [0.96,1.42]	1.74 [1.42,2.14]	1.66 [1.35,2.04]
	Other essential/key workers	1.09 [0.90,1.32]	1.04 [0.85,1.26]	1.24 [1.09,1.42]	1.16 [1.02,1.33]	1.19 [1.02,1.39]	1.18 [1.01,1.37]
	Other worker	Ref	Ref	Ref	Ref	Ref	Ref
	Not FT, PT, SE*	1.09 [0.91,1.32]	1.04 [0.86,1.25]	0.97 [0.85,1.10]	0.93 [0.82,1.06]	0.94 [0.80,1.10]	0.91 [0.78,1.07]
	Unknown	1.07 [0.69,1.65]	0.98 [0.63,1.51]	1.18 [0.88,1.59]	1.10 [0.82,1.48]	1.48 [1.12,1.95]	1.39 [1.05,1.84]
Deprivation Index Quintile	1 - Most Deprived	1.60 [1.29,1.98]	1.10 [0.88,1.38]	1.66 [1.42,1.95]	1.25 [1.06,1.47]	1.89 [1.58,2.27]	1.70 [1.41,2.05]
	2	1.00 [0.81,1.25]	0.86 [0.69,1.07]	1.20 [1.03,1.39]	1.08 [0.92,1.25]	1.41 [1.19,1.68]	1.34 [1.13,1.60]
	3	0.85 [0.69,1.05]	0.80 [0.65,0.99]	1.08 [0.94,1.25]	1.04 [0.90,1.20]	1.19 [1.01,1.40]	1.17 [0.99,1.38]
	4	0.97 [0.80,1.18]	0.91 [0.75,1.10]	1.08 [0.95,1.24]	1.02 [0.89,1.17]	1.11 [0.94,1.30]	1.09 [0.93,1.28]
	5 - Least Deprived	Ref	Ref		Ref		Ref
Ethnicity	English/Welsh/Scottish/Northern Irish/British	Ref	Ref	Ref	Ref	Ref	Ref
	Irish	0.84 [0.38,1.89]	0.95 [0.42,2.13]	1.07 [0.65,1.76]	1.25 [0.76,2.05]	0.48 [0.20,1.16]	0.49 [0.20,1.19]
	Gypsy or Irish Traveller	NA	NA	NA	NA	2.45 [0.34,17.84]	2.55 [0.35,18.71]
	Other white background	0.80 [0.56,1.14]	0.97 [0.67,1.39]	1.00 [0.80,1.25]	1.23 [0.98,1.53]	1.02 [0.79,1.32]	1.06 [0.82,1.38]
	White and Black Caribbean	0.58 [0.14,2.34]	0.64 [0.16,2.58]	1.41 [0.73,2.74]	1.45 [0.75,2.82]	0.90 [0.37,2.19]	0.85 [0.35,2.07]
	White and Black African	NA	NA	0.61 [0.15,2.48]	0.68 [0.17,2.76]	0.31 [0.04,2.18]	0.29 [0.04,2.07]
	White and Asian	0.35 [0.09,1.43]	0.39 [0.10,1.59]	0.81 [0.42,1.56]	0.93 [0.48,1.81]	1.09 [0.60,1.99]	1.15 [0.63,2.11]
	Other Mixed/Multiple ethnic background	1.35 [0.64,2.86]	1.60 [0.75,3.40]	0.73 [0.36,1.47]	0.85 [0.42,1.72]	0.67 [0.30,1.51]	0.72 [0.32,1.61]
	Indian	1.37 [0.92,2.03]	1.63 [1.09,2.44]	0.92 [0.65,1.29]	1.07 [0.76,1.52]	1.53 [1.12,2.09]	1.52 [1.11,2.09]
	Pakistani	2.77 [1.65,4.66]	2.57 [1.52,4.35]	2.15 [1.38,3.37]	2.05 [1.30,3.22]	2.53 [1.56,4.13]	2.29 [1.40,3.74]
	Bangladeshi	5.17 [3.01,8.88]	5.59 [3.20,9.78]	1.50 [0.67,3.38]	1.77 [0.78,4.00]	4.64 [2.79,7.71]	4.32 [2.57,7.27]
	Chinese	0.68 [0.22,2.11]	0.78 [0.25,2.45]	0.49 [0.20,1.19]	0.58 [0.24,1.40]	0.45 [0.14,1.40]	0.49 [0.16,1.52]
	Other Asian background	1.57 [0.91,2.73]	1.93 [1.10,3.38]	1.19 [0.76,1.88]	1.48 [0.93,2.34]	1.35 [0.83,2.19]	1.33 [0.81,2.16]
	African	1.82 [1.05,3.16]	1.98 [1.13,3.49]	1.31 [0.81,2.13]	1.47 [0.90,2.40]	1.62 [0.98,2.67]	1.40 [0.84,2.33]

Caribbean	1.12 [0.36,3.49]	1.36 [0.43,4.28]	1.18 [0.53,2.66]	1.34 [0.60,3.02]	0.53 [0.13,2.12]	0.48 [0.12,1.94]
Other Black/African/Caribbean background	NA	NA	NA	NA	NA	NA
Arab	2.26 [0.84,6.07]	2.30 [0.85,6.22]	0.87 [0.28,2.73]	0.93 [0.30,2.92]	1.64 [0.61,4.42]	1.60 [0.59,4.33]
Other ethnic group	0.58 [0.19,1.82]	0.68 [0.22,2.14]	1.38 [0.81,2.35]	1.67 [0.98,2.85]	1.63 [0.92,2.90]	1.67 [0.94,2.97]
Prefer not to say	0.84 [0.35,2.03]	0.91 [0.37,2.19]	1.37 [0.84,2.21]	1.53 [0.94,2.48]	1.80 [1.08,3.01]	1.80 [1.07,3.01]
Unknown	1.33 [0.63,2.82]	1.35 [0.64,2.87]	1.08 [0.63,1.88]	1.07 [0.61,1.85]	1.25 [0.67,2.33]	1.18 [0.63,2.20]

* HCW/CHW: health care worker/care home worker; Not FT, PT SE: not self-employed, full-time, part-time, self-employed.

Round 8		Round 9		Round 10		Rounds 5 to 10	
Adjusted for age and sex	Mutually adjusted	Adjusted for age and sex	Mutually adjusted	Adjusted for age and sex	Mutually adjusted	Adjusted for age and sex	Mutually adjusted
Ref	Ref	Ref	Ref	Ref	Ref	Ref	Ref
0.94 [0.87,1.02]	0.93 [0.86,1.01]	0.97 [0.83,1.12]	0.96 [0.82,1.12]	0.87 [0.67,1.14]	0.88 [0.67,1.15]	0.95 [0.91, 1.00]	0.95 [0.90, 0.99]
1.00 [0.83,1.21]	1.01 [0.84,1.22]	1.33 [0.99,1.80]	1.38 [1.02,1.86]	1.50 [0.93,2.42]	1.48 [0.91,2.39]	1.15 [0.93, 1.43]	1.16 [0.94, 1.45]
1.33 [1.11,1.61]	1.71 [1.39,2.11]	0.99 [0.70,1.42]	1.12 [0.76,1.65]	0.87 [0.47,1.63]	0.79 [0.40,1.57]	1.3 [0.99, 1.71]	1.41 [1.04, 1.92]
1.62 [1.33,1.96]	1.73 [1.43,2.11]	1.33 [0.91,1.92]	1.40 [0.96,2.04]	1.11 [0.59,2.11]	1.09 [0.57,2.08]	1.73 [1.49, 2.01]	1.8 [1.61, 2.01]
1.22 [1.03,1.44]	1.19 [1.01,1.41]	1.03 [0.75,1.41]	1.01 [0.74,1.38]	0.86 [0.50,1.47]	0.83 [0.48,1.42]	1.18 [1.07, 1.3]	1.16 [1.06, 1.28]
Ref	Ref	Ref	Ref	Ref	Ref	Ref	Ref
1.02 [0.88,1.18]	1.08 [0.93,1.25]	1.07 [0.82,1.40]	1.11 [0.85,1.45]	0.82 [0.52,1.30]	0.83 [0.52,1.33]	1.05 [0.96, 1.14]	1.08 [0.99, 1.17]
0.89 [0.77,1.03]	1.02 [0.88,1.18]	0.95 [0.73,1.24]	1.04 [0.79,1.36]	0.74 [0.47,1.17]	0.77 [0.48,1.22]	0.87 [0.75, 1]	0.92 [0.8, 1.06]
0.60 [0.52,0.70]	0.80 [0.67,0.94]	0.53 [0.40,0.70]	0.64 [0.46,0.87]	0.44 [0.28,0.71]	0.48 [0.29,0.81]	0.6 [0.53, 0.69]	0.71 [0.63, 0.81]
0.74 [0.57,0.96]	0.68 [0.52,0.89]	2.20 [1.54,3.14]	1.89 [1.32,2.72]	5.75 [2.93,11.28]	5.28 [2.67,10.46]	2.11 [1.22, 3.65]	1.95 [1.11, 3.41]
0.84 [0.72,0.98]	0.78 [0.66,0.91]	1.81 [1.40,2.34]	1.60 [1.23,2.09]	4.51 [2.59,7.85]	4.18 [2.38,7.34]	2.16 [1.28, 3.65]	2.01 [1.16, 3.48]
0.60 [0.48,0.74]	0.57 [0.45,0.71]	1.17 [0.82,1.67]	1.09 [0.76,1.55]	5.16 [2.85,9.33]	5.00 [2.75,9.08]	1.77 [0.98, 3.17]	1.7 [0.94, 3.1]
0.71 [0.60,0.82]	0.68 [0.58,0.79]	1.51 [1.16,1.97]	1.44 [1.10,1.88]	3.33 [1.88,5.89]	3.27 [1.85,5.81]	1.53 [1.06, 2.23]	1.5 [1.02, 2.19]
0.89 [0.76,1.05]	0.85 [0.73,1.01]	1.20 [0.88,1.64]	1.11 [0.81,1.52]	3.53 [1.93,6.44]	3.41 [1.86,6.23]	1.55 [1.12, 2.16]	1.49 [1.06, 2.09]
1.05 [0.92,1.20]	1.03 [0.90,1.17]	1.12 [0.85,1.48]	1.09 [0.83,1.45]	2.35 [1.29,4.27]	2.31 [1.27,4.19]	1.06 [0.87, 1.3]	1.05 [0.86, 1.3]
1.62 [1.42,1.84]	1.43 [1.24,1.64]	1.73 [1.32,2.28]	1.52 [1.14,2.02]	2.52 [1.35,4.70]	2.16 [1.14,4.10]	1.51 [1.31, 1.73]	1.34 [1.17, 1.52]
Ref	Ref	Ref	Ref	Ref	Ref	Ref	Ref
0.52 [0.42,0.63]	0.50 [0.41,0.61]	0.66 [0.46,0.97]	0.65 [0.44,0.94]	1.90 [0.95,3.76]	1.89 [0.95,3.77]	0.93 [0.65, 1.32]	0.92 [0.64, 1.34]
1.47 [1.24,1.74]	1.48 [1.25,1.77]	1.42 [1.06,1.92]	1.37 [1.02,1.84]	1.21 [0.73,1.99]	1.09 [0.66,1.81]	1.41 [1.24, 1.6]	1.32 [1.13, 1.54]
1.28 [1.15,1.43]	1.36 [1.22,1.52]	1.22 [1.00,1.49]	1.20 [0.99,1.47]	0.98 [0.68,1.41]	0.90 [0.62,1.29]	1.22 [1.14, 1.3]	1.18 [1.07, 1.3]
Ref	Ref	Ref	Ref	Ref	Ref	Ref	Ref
0.91 [0.81,1.02]	0.93 [0.82,1.04]	0.99 [0.80,1.22]	0.95 [0.77,1.18]	0.95 [0.66,1.36]	0.89 [0.62,1.28]	0.96 [0.9, 1.02]	0.94 [0.88, 1]
0.76 [0.57,1.03]	0.76 [0.57,1.02]	1.11 [0.71,1.73]	1.05 [0.67,1.64]	1.79 [0.88,3.64]	1.61 [0.79,3.28]	1.14 [0.91, 1.44]	1.08 [0.87, 1.33]
1.41 [1.22,1.64]	1.41 [1.21,1.65]	1.95 [1.52,2.49]	1.67 [1.29,2.15]	2.00 [1.31,3.06]	1.44 [0.93,2.23]	1.69 [1.51, 1.89]	1.41 [1.22, 1.62]
1.47 [1.29,1.66]	1.39 [1.22,1.58]	1.62 [1.29,2.03]	1.48 [1.17,1.86]	1.57 [1.05,2.33]	1.35 [0.91,2.02]	1.34 [1.17, 1.54]	1.22 [1.04, 1.44]
1.15 [1.01,1.30]	1.14 [1.00,1.29]	1.01 [0.79,1.27]	0.98 [0.77,1.24]	1.10 [0.74,1.64]	1.01 [0.67,1.50]	1.07 [0.98, 1.18]	1.03 [0.92, 1.16]
1.12 [0.99,1.26]	1.13 [1.00,1.28]	1.12 [0.90,1.40]	1.09 [0.88,1.37]	1.05 [0.71,1.56]	0.96 [0.65,1.43]	1.09 [1.02, 1.16]	1.06 [0.98, 1.13]
	Ref		Ref		Ref		Ref
Ref	Ref	Ref	Ref	Ref	Ref	Ref	Ref
1.63 [1.13,2.35]	1.41 [0.98,2.04]	1.17 [0.52,2.62]	1.16 [0.52,2.61]	0.52 [0.07,3.68]	0.57 [0.08,4.12]	1.05 [0.72, 1.53]	1.14 [0.86, 1.51]
NA	NA	NA	NA	0.00 [0.00,Inf]	0.00 [0.00,Inf]	2.44 [0.33, 17.72]	2.54 [0.35, 18.57]
1.49 [1.26,1.76]	1.25 [1.05,1.49]	2.02 [1.56,2.62]	2.01 [1.54,2.64]	1.11 [0.64,1.93]	1.29 [0.73,2.27]	1.2 [0.91, 1.58]	1.27 [1.03, 1.57]
0.98 [0.46,2.06]	0.82 [0.39,1.74]	0.42 [0.06,3.01]	0.39 [0.06,2.81]	2.07 [0.51,8.42]	2.09 [0.51,8.53]	1.08 [0.73, 1.59]	1.03 [0.70, 1.52]
1.23 [0.51,2.99]	1.05 [0.43,2.55]	NA	NA	1.68 [0.23,12.11]	1.76 [0.24,12.75]	0.93 [0.48, 1.80]	0.87 [0.45, 1.69]
1.28 [0.80,2.04]	1.15 [0.72,1.84]	1.05 [0.43,2.55]	1.08 [0.44,2.62]	1.09 [0.27,4.43]	1.30 [0.32,5.29]	1.04 [0.78, 1.38]	1.05 [0.79, 1.41]
1.19 [0.72,1.96]	1.01 [0.61,1.67]	0.47 [0.12,1.88]	0.46 [0.11,1.86]	1.30 [0.32,5.28]	1.51 [0.37,6.16]	0.97 [0.72, 1.33]	0.99 [0.72, 1.34]
1.63 [1.28,2.09]	1.36 [1.06,1.74]	2.06 [1.39,3.07]	2.02 [1.35,3.02]	1.02 [0.42,2.48]	1.13 [0.46,2.78]	1.42 [1.12, 1.8]	1.44 [1.22, 1.71]
1.86 [1.18,2.95]	1.58 [0.99,2.50]	3.21 [1.75,5.86]	2.68 [1.46,4.93]	2.11 [0.67,6.64]	1.81 [0.57,5.77]	2.37 [1.91, 2.95]	2.12 [1.70, 2.64]
4.40 [2.79,6.95]	3.03 [1.90,4.82]	1.96 [0.63,6.15]	1.57 [0.50,4.96]	3.05 [0.75,12.39]	2.87 [0.69,11.89]	3.60 [2.45, 5.29]	3.28 [2.24, 4.80]
0.82 [0.42,1.58]	0.70 [0.36,1.36]	0.33 [0.05,2.34]	0.33 [0.05,2.34]	0.78 [0.11,5.59]	0.84 [0.12,6.03]	0.63 [0.41, 0.95]	0.63 [0.41, 0.96]
2.33 [1.73,3.13]	1.81 [1.34,2.45]	1.83 [1.00,3.33]	1.72 [0.94,3.16]	1.29 [0.41,4.04]	1.44 [0.45,4.58]	1.64 [1.25, 2.15]	1.66 [1.36, 2.01]
2.78 [2.03,3.80]	1.92 [1.39,2.65]	1.85 [1.01,3.37]	1.46 [0.79,2.70]	1.65 [0.61,4.46]	1.54 [0.56,4.28]	1.87 [1.42, 2.48]	1.69 [1.38, 2.07]

1.72 [0.97,3.06]	1.24 [0.70,2.22]	0.44 [0.06,3.12]	0.39 [0.05,2.76]	NA	NA	1.24 [0.81, 1.91]	1.12 [0.75, 1.69]
3.02 [1.33,6.85]	2.25 [0.99,5.12]	3.35 [0.83,13.58]	2.93 [0.72,11.94]	NA	NA	3.10 [1.53, 6.28]	2.40 [1.18, 4.89]
1.49 [0.66,3.34]	1.18 [0.52,2.66]	3.07 [1.14,8.26]	2.69 [0.99,7.28]	1.89 [0.26,13.57]	1.88 [0.26,13.66]	1.75 [1.15, 2.67]	1.62 [1.06, 2.47]
1.93 [1.27,2.93]	1.51 [0.99,2.29]	0.55 [0.14,2.20]	0.52 [0.13,2.09]	1.33 [0.33,5.38]	1.46 [0.36,5.94]	1.48 [1.11, 1.98]	1.45 [1.11, 1.9]
1.55 [1.06,2.25]	1.36 [0.93,1.99]	1.58 [0.75,3.34]	1.54 [0.72,3.25]	0.65 [0.09,4.65]	0.68 [0.09,4.87]	1.47 [1.16, 1.86]	1.44 [1.14, 1.82]
0.96 [0.60,1.54]	0.93 [0.58,1.49]	1.12 [0.50,2.52]	1.08 [0.48,2.42]	2.16 [0.80,5.86]	2.10 [0.77,5.68]	1.16 [0.89, 1.50]	1.12 [0.87, 1.46]
