# Supplementary material for: Health inequalities in risk of SARS-CoV-2 infection in England’s second wave: population-based cross-sectional analysis from the REACT-1 study
Source: BMJ Public Health. 2026 Jan 5;4(1):e002596. doi: 10.1136/bmjph-2025-002596 (PMC13663695; doi:10.1136/bmjph-2025-002596)
Supplement: Supplementary file 7 [file bmjph-4-1-s007.pdf]

Supplementary Table 6. The unweighted and weighted prevalence of swab-positivity for detailed categories of occupation for round 5.

Variable	Category	Round 5				Round 6				Round 7			
		Positive	Total	Unweighted Prevalence	Weighted Prevalence*	Positive	Total	Unweighted Prevalence	Weighted Prevalence*	Positive	Total	Unweighted Prevalence	Weighted Prevalence*
Work type 1**	1. Health care workers with direct patient contact	47	6,852	0.69% (0.52% , 0.91%)	0.94% (0.62% , 1.42%)	89	6,755	1.32% (1.14% , 1.70%)	1.75% (1.21% , 2.52%)	96	6,892	1.39% (1.14% , 1.70%)	1.62% (1.25% , 2.10%)
	2. Health care workers with no patient contact	7	2,100	0.33% (0.16% , 0.69%)	NA (NA , NA)	18	1,999	0.90% (0.56% , 1.39%)	1.05% (0.61% , 1.82%)	18	2,043	0.88% (0.56% , 1.39%)	1.33% (0.73% , 2.44%)
	3. Care home workers with direct contact with clients	4	926	0.43% (0.17% , 1.11%)	NA (NA , NA)	17	820	2.07% (0.86% , 2.60%)	2.90% (1.57% , 5.30%)	12	800	1.50% (0.86% , 2.60%)	1.80% (0.84% , 3.83%)
	4. Care home workers without contact with clients	1	171	0.58% (0.10% , 3.24%)	NA (NA , NA)	3	161	1.86% (0.31% , 3.96%)	NA (NA , NA)	2	180	1.11% (0.31% , 3.96%)	NA (NA , NA)
	5. Other essential/key workers	155	29,639	0.52% (0.45% , 0.61%)	0.68% (0.54% , 0.86%)	366	27,700	1.32% (0.80% , 1.01%)	1.63% (1.40% , 1.90%)	279	30,907	0.90% (0.80% , 1.01%)	1.07% (0.91% , 1.25%)
	6. None of these	556	126,026	0.44% (0.41% , 0.48%)	0.56% (0.50% , 0.63%)	1121	115,136	0.97% (0.58% , 0.67%)	1.15% (1.05% , 1.25%)	744	118,941	0.63% (0.58% , 0.67%)	0.77% (0.69% , 0.85%)
	7. Don't know	15	2,467	0.61% (0.37% , 1.00%)	0.83% (0.43% , 1.60%)	37	2,365	1.56% (0.81% , 1.67%)	1.49% (0.98% , 2.26%)	28	2,409	1.16% (0.81% , 1.67%)	1.13% (0.73% , 1.76%)
	NA	33	6,449	0.51% (0.36% , 0.72%)	0.65% (0.39% , 1.10%)	80	5,237	1.53% (1.67% , 2.38%)	1.82% (1.36% , 2.44%)	120	6,009	2.00% (1.67% , 2.38%)	2.37% (1.88% , 2.98%)
Work type 2**	1. Delivering to homes - No	718	156,374	0.46% (0.43% , 0.49%)	0.59% (0.53% , 0.65%)	1508	143,585	1.05% (0.65% , 0.73%)	1.25% (1.16% , 1.34%)	1036	150,558	0.69% (0.65% , 0.73%)	0.83% (0.76% , 0.90%)
	Delivering to homes - Yes	8	1,758	0.46% (0.23% , 0.90%)	NA (NA , NA)	16	1,616	0.99% (0.54% , 1.45%)	1.64% (0.77% , 3.48%)	15	1,699	0.88% (0.54% , 1.45%)	1.46% (0.73% , 2.89%)
	2. Food retail, other shop work - No	677	150,874	0.45% (0.42% , 0.48%)	0.56% (0.51% , 0.62%)	1426	138,899	1.03% (0.64% , 0.72%)	1.21% (1.12% , 1.30%)	989	146,228	0.68% (0.64% , 0.72%)	0.82% (0.75% , 0.89%)
	Food retail, other shop work - Yes	49	7,258	0.68% (0.51% , 0.89%)	1.05% (0.65% , 1.71%)	98	6,302	1.56% (0.80% , 1.32%)	2.04% (1.44% , 2.88%)	62	6,029	1.03% (0.80% , 1.32%)	1.26% (0.89% , 1.78%)
	10. Hospitality - No	694	154,203	0.45% (0.42% , 0.48%)	0.56% (0.51% , 0.62%)	1474	141,870	1.04% (0.64% , 0.72%)	1.23% (1.14% , 1.33%)	1009	149,328	0.68% (0.64% , 0.72%)	0.82% (0.75% , 0.90%)
	Hospitality - Yes	32	3,929	0.81% (0.58% , 1.15%)	1.41% (0.87% , 2.26%)	50	3,331	1.50% (1.06% , 1.93%)	1.84% (1.30% , 2.59%)	42	2,929	1.43% (1.06% , 1.93%)	1.56% (1.05% , 2.30%)
	11. Personal care - No	721	156,518	0.46% (0.43% , 0.50%)	0.59% (0.53% , 0.65%)	1502	143,911	1.04% (1.07% , 1.62%)	1.24% (1.15% , 1.34%)	1040	150,953	0.69% (0.65% , 0.73%)	0.84% (0.77% , 0.91%)
	Personal care - Yes	5	1,614	0.31% (0.13% , 0.72%)	NA (NA , NA)	22	1,290	1.71% (0.57% , 1.42%)	2.16% (1.19% , 3.87%)	11	1,304	0.84% (0.47% , 1.50%)	0.90% (0.43% , 1.86%)
	3. Policing, prisons, fire & rescue, coastguard - No	720	156,764	0.46% (0.43% , 0.49%)	0.59% (0.53% , 0.65%)	1508	143,812	1.05% (1.19% , 1.46%)	1.25% (1.16% , 1.35%)	1031	150,919	0.68% (0.64% , 0.73%)	0.83% (0.77% , 0.91%)
	Policing, prisons, fire & rescue, coastguard - Yes	6	1,368	0.44% (0.20% , 0.95%)	NA (NA , NA)	16	1,389	1.15% (0.92% , 1.03%)	1.17% (0.67% , 2.05%)	20	1,338	1.49% (0.97% , 2.30%)	1.54% (0.93% , 2.55%)
	4. Public transport - No	716	156,742	0.46% (0.42% , 0.49%)	0.58% (0.52% , 0.65%)	1514	144,024	1.05% (1.00% , 1.10%)	1.25% (1.16% , 1.35%)	1039	151,195	0.69% (0.65% , 0.73%)	0.84% (0.77% , 0.91%)
	Public transport - Yes	10	1,390	0.72% (0.39% , 1.32%)	0.88% (0.44% , 1.78%)	10	1,177	0.85% (0.61% , 1.60%)	0.96% (0.47% , 1.98%)	12	1,062	1.13% (0.65% , 1.96%)	1.05% (0.55% , 2.01%)
	5. Education, school, nursery or childcare - No	655	145,156	0.45% (0.42% , 0.49%)	0.59% (0.53% , 0.65%)	1358	132,949	1.02% (0.98% , 1.08%)	1.23% (1.13% , 1.33%)	937	139,745	0.67% (0.63% , 0.71%)	0.83% (0.76% , 0.91%)
	Education, school, nursery or childcare - Yes	71	12,976	0.55% (0.43% , 0.69%)	0.58% (0.43% , 0.79%)	166	12,252	1.35% (1.28% , 1.89%)	1.50% (1.21% , 1.84%)	114	12,512	0.91% (0.76% , 1.09%)	0.95% (0.75% , 1.19%)
	12. Childcare - No	NA	NA	NA (NA , NA)	NA (NA , NA)	NA	NA	NA (NA , NA)	NA (NA , NA)	NA	NA	NA (NA , NA)	NA (NA , NA)
	Childcare - Yes	NA	NA	NA (NA , NA)	NA (NA , NA)	NA	NA	NA (NA , NA)	NA (NA , NA)	NA	NA	NA (NA , NA)	NA (NA , NA)
	6. Armed forces - No	726	157,911	0.46% (0.43% , 0.49%)	0.58% (0.52% , 0.65%)	1524	144,999	1.05% (1.00% , 1.10%)	1.25% (1.16% , 1.35%)	1050	152,061	0.69% (0.65% , 0.73%)	0.91% (0.84% , 0.77%)
	Armed forces - Yes	0	221	0.00% (0.00% , 1.21%)	NA (NA , NA)	0	202	0.00% (0.00% , 1.32%)	NA (NA , NA)	1	196	0.51% (0.09% , 2.83%)	NA (NA , NA)
	7. Another public facing role - No	658	143,212	0.46% (0.43% , 0.50%)	0.58% (0.52% , 0.65%)	1352	131,809	1.03% (0.97% , 1.08%)	1.21% (1.12% , 1.31%)	943	139,442	0.68% (0.63% , 0.72%)	0.91% (0.82% , 0.74%)
	Another public facing role - Yes	68	14,920	0.46% (0.36% , 0.58%)	0.59% (0.48% , 0.74%)	172	13,392	1.28% (1.11% , 1.49%)	1.60% (1.31% , 1.96%)	108	12,815	0.84% (0.70% , 1.02%)	1.17% (1.06% , 0.81%)
	8. Work outside but not in public facing role - No	595	133,428	0.45% (0.41% , 0.48%)	0.70% (0.60% , 0.81%)	1267	123,069	1.03% (0.97% , 1.09%)	1.23% (1.14% , 1.34%)	893	131,410	0.68% (0.64% , 0.73%)	0.89% (0.82% , 0.74%)
	Work outside but not in public facing role - Yes	131	24,704	0.53% (0.45% , 0.63%)	0.50% (0.43% , 0.57%)	257	22,132	1.16% (1.03% , 1.31%)	1.34% (1.14% , 1.57%)	158	20,847	0.76% (0.65% , 0.89%)	1.25% (1.01% , 0.75%)
	9. Not currently required to work outside my home - No	366	67,291	0.54% (0.49% , 0.60%)	1.53% (1.38% , 1.69%)	782	60,489	1.29% (1.21% , 1.39%)	1.53% (1.38% , 1.69%)	523	58,269	0.90% (0.82% , 0.98%)	1.20% (1.10% , 0.95%)
	Not currently required to work outside my home-Yes	360	90,841	0.40% (0.36% , 0.44%)	1.04% (0.93% , 1.16%)	742	84,712	0.88% (0.82% , 0.94%)	1.04% (0.93% , 1.16%)	528	93,988	0.56% (0.52% , 0.61%)	0.78% (0.68% , 0.60%)

* Work type 1 and 2 were asked respondents/their children

** Respondents only answered for work type 2 if they selected An essential/ key workers, /None of these/Don't know for work type 1.

Round 8				Round 9				Round 10			
Positive	Total	Unweighted Prevalence	Weighted Prevalence*	Positive	Total	Unweighted Prevalence	Weighted Prevalence*	Positive	Total	Unweighted Prevalence	Weighted Prevalence*
124	5,922	2.09% (1.76% , 2.49%)	2.43% (1.93% , 3.05%)	42	6,430	0.65% (0.48% , 0.88%)	0.87% (0.55% , 1.37%)	15	6,190	0.24% (0.15% , 0.40%)	0.24% (0.13% , 0.43%)
14	1,896	0.74% (0.44% , 1.24%)	0.97% (0.49% , 1.92%)	9	2,041	0.44% (0.23% , 0.84%)	NA (NA , NA)	2	1,895	0.11% (0.03% , 0.38%)	NA (NA , NA)
28	780	3.59% (2.50% , 5.14%)	4.42% (2.86% , 6.77%)	4	891	0.45% (0.17% , 1.15%)	NA (NA , NA)	2	850	0.24% (0.06% , 0.85%)	NA (NA , NA)
9	156	5.77% (3.06% , 10.60%)	NA (NA , NA)	3	198	1.52% (0.52% , 4.36%)	NA (NA , NA)	0	182	0.00% (0.00% , 1.46%)	NA (NA , NA)
552	31,300	1.76% (1.62% , 1.92%)	1.84% (1.66% , 2.05%)	168	32,426	0.52% (0.45% , 0.60%)	0.55% (0.45% , 0.68%)	46	24,898	0.18% (0.14% , 0.25%)	0.21% (0.14% , 0.30%)
1430	119,720	1.19% (1.13% , 1.26%)	1.41% (1.31% , 1.51%)	411	112,869	0.36% (0.33% , 0.40%)	0.45% (0.39% , 0.51%)	147	100,253	0.15% (0.12% , 0.17%)	0.19% (0.15% , 0.23%)
33	2,479	1.33% (0.95% , 1.86%)	1.43% (0.94% , 2.17%)	11	2,309	0.48% (0.27% , 0.85%)	0.43% (0.20% , 0.92%)	6	1,910	0.31% (0.14% , 0.68%)	NA (NA , NA)
92	5,389	1.71% (1.39% , 2.09%)	2.02% (1.54% , 2.63%)	41	8,292	0.49% (0.36% , 0.67%)	0.66% (0.43% , 1.01%)	9	4,666	0.19% (0.10% , 0.37%)	NA (NA , NA)
1985	151,649	1.31% (1.25% , 1.37%)	1.49% (1.41% , 1.59%)	584	145,767	0.40% (0.37% , 0.43%)	0.47% (0.42% , 0.53%)	194	125,597	0.15% (0.13% , 0.18%)	0.19% (0.16% , 0.23%)
30	1,850	1.62% (1.14% , 2.31%)	2.09% (1.24% , 3.50%)	6	1,837	0.33% (0.15% , 0.71%)	NA (NA , NA)	5	1,464	0.34% (0.15% , 0.80%)	NA (NA , NA)
1886	146,737	1.29% (1.23% , 1.34%)	1.46% (1.37% , 1.54%)	561	142,123	0.39% (0.36% , 0.43%)	0.46% (0.41% , 0.52%)	189	122,364	0.15% (0.13% , 0.18%)	0.19% (0.16% , 0.23%)
129	6,762	1.91% (1.61% , 2.26%)	2.36% (1.75% , 3.19%)	29	5,481	0.53% (0.37% , 0.76%)	0.66% (0.40% , 1.11%)	10	4,697	0.21% (0.12% , 0.39%)	0.29% (0.13% , 0.61%)
1957	150,372	1.30% (1.25% , 1.36%)	1.49% (1.40% , 1.58%)	581	145,588	0.40% (0.37% , 0.43%)	0.47% (0.42% , 0.52%)	198	124,984	0.16% (0.14% , 0.18%)	0.20% (0.17% , 0.24%)
58	3,127	1.85% (1.44% , 2.39%)	2.16% (1.59% , 2.95%)	9	2,016	0.45% (0.24% , 0.85%)	NA (NA , NA)	1	2,077	0.05% (0.01% , 0.27%)	NA (NA , NA)
1998	151,998	1.31% (1.26% , 1.37%)	1.50% (1.41% , 1.59%)	588	146,632	0.40% (0.37% , 0.43%)	0.47% (0.42% , 0.53%)	198	126,136	0.16% (0.14% , 0.18%)	0.20% (0.17% , 0.24%)
17	1,501	1.13% (0.71% , 1.81%)	1.94% (1.09% , 3.43%)	2	972	0.21% (0.06% , 0.75%)	NA (NA , NA)	1	925	0.11% (0.02% , 0.61%)	NA (NA , NA)
1984	152,134	1.30% (1.25% , 1.36%)	1.49% (1.41% , 1.58%)	581	146,221	0.40% (0.37% , 0.43%)	0.47% (0.42% , 0.52%)	196	126,100	0.16% (0.14% , 0.18%)	0.20% (0.16% , 0.24%)
31	1,365	2.27% (1.60% , 3.21%)	2.51% (1.60% , 3.91%)	9	1,383	0.65% (0.34% , 1.23%)	NA (NA , NA)	3	961	0.31% (0.11% , 0.91%)	NA (NA , NA)
1973	152,277	1.30% (1.24% , 1.35%)	1.48% (1.40% , 1.57%)	578	146,467	0.39% (0.36% , 0.43%)	0.46% (0.41% , 0.52%)	196	126,064	0.16% (0.14% , 0.18%)	0.20% (0.16% , 0.23%)
42	1,222	3.44% (2.55% , 4.61%)	3.70% (2.53% , 5.38%)	12	1,137	1.06% (0.60% , 1.84%)	1.42% (0.72% , 2.77%)	3	997	0.30% (0.10% , 0.88%)	NA (NA , NA)
1811	141,182	1.28% (1.23% , 1.34%)	1.47% (1.38% , 1.56%)	538	139,192	0.39% (0.36% , 0.42%)	0.46% (0.41% , 0.51%)	182	118,799	0.15% (0.13% , 0.18%)	0.19% (0.16% , 0.23%)
204	12,317	1.66% (1.45% , 1.90%)	1.85% (1.54% , 2.24%)	52	8,412	0.62% (0.47% , 0.81%)	0.73% (0.52% , 1.02%)	17	8,262	0.21% (0.13% , 0.33%)	0.27% (0.15% , 0.48%)
NA	NA	NA (NA , NA)	NA (NA , NA)	588	146,778	0.40% (0.37% , 0.43%)	0.47% (0.42% , 0.53%)	197	126,286	0.16% (0.14% , 0.18%)	0.20% (0.16% , 0.23%)
NA	NA	NA (NA , NA)	NA (NA , NA)	2	826	0.24% (0.07% , 0.88%)	NA (NA , NA)	2	775	0.26% (0.07% , 0.94%)	NA (NA , NA)
2010	153,285	1.31% (1.26% , 1.37%)	1.50% (1.42% , 1.59%)	588	147,398	0.40% (0.37% , 0.43%)	0.47% (0.42% , 0.53%)	199	126,916	0.16% (0.14% , 0.18%)	0.20% (0.16% , 0.23%)
5	214	2.34% (1.00% , 5.35%)	NA (NA , NA)	2	206	0.97% (0.27% , 3.47%)	NA (NA , NA)	0	145	0.00% (0.00% , 1.83%)	NA (NA , NA)
1793	139,688	1.28% (1.23% , 1.34%)	1.47% (1.38% , 1.56%)	529	135,827	0.39% (0.36% , 0.42%)	0.46% (0.41% , 0.51%)	182	116,983	0.16% (0.13% , 0.18%)	0.19% (0.16% , 0.23%)
222	13,811	1.61% (1.41% , 1.83%)	1.84% (1.55% , 2.17%)	61	11,777	0.52% (0.40% , 0.66%)	0.61% (0.43% , 0.87%)	17	10,078	0.17% (0.11% , 0.27%)	0.23% (0.12% , 0.46%)
1686	131,481	1.28% (1.22% , 1.34%)	1.51% (1.41% , 1.61%)	481	129,247	0.37% (0.34% , 0.41%)	0.45% (0.40% , 0.51%)	165	111,864	0.15% (0.13% , 0.17%)	0.19% (0.16% , 0.23%)
329	22,018	1.49% (1.34% , 1.66%)	1.48% (1.30% , 1.68%)	109	18,357	0.59% (0.49% , 0.72%)	0.63% (0.49% , 0.81%)	34	15,197	0.22% (0.16% , 0.31%)	0.24% (0.16% , 0.36%)
1035	61,457	1.68% (1.59% , 1.79%)	1.90% (1.75% , 2.07%)	281	50,148	0.56% (0.50% , 0.63%)	0.64% (0.55% , 0.75%)	87	43,605	0.20% (0.16% , 0.25%)	0.24% (0.19% , 0.32%)
980	92,042	1.06% (1.00% , 1.13%)	1.22% (1.12% , 1.32%)	309	97,456	0.32% (0.28% , 0.35%)	0.38% (0.33% , 0.44%)	112	83,456	0.13% (0.11% , 0.16%)	0.17% (0.13% , 0.22%)
