# Supplementary material for: Health inequalities in risk of SARS-CoV-2 infection in England’s second wave: population-based cross-sectional analysis from the REACT-1 study
Source: BMJ Public Health. 2026 Jan 5;4(1):e002596. doi: 10.1136/bmjph-2025-002596 (PMC13663695; doi:10.1136/bmjph-2025-002596)
Supplement: Supplementary file 8 [file bmjph-4-1-s008.pdf]

**Supplementary Table 7.** Odds ratios for rounds 5 to 10, and an average weighted inversely to variance for detailed subgroups of occupations.

| Variable | Category | Round 5 | | Round 6 | | Round 7 | | Round 8 | |
|---|---|---|---|---|---|---|---|---|---|
| | | Adjusted for age and sex | Mutually adjusted | Adjusted for age and sex | Mutually adjusted | Adjusted for age and sex | Mutually adjusted | Adjusted for age and sex | Mutually adjusted |
| Gender | Male | Ref | Ref | Ref | Ref | Ref | Ref | Ref | Ref |
| | Female | 0.90 [0.78,1.03] | 0.90 [0.78,1.03] | 1.01 [0.92,1.11] | 1.01 [0.91,1.11] | 0.95 [0.85,1.06] | 0.93 [0.83,1.04] | 0.94 [0.87,1.02] | 0.93 [0.86,1.01] |
| Age Group | 5-12 | 0.80 [0.59,1.10] | 0.81 [0.59,1.10] | 0.99 [0.78,1.24] | 1.00 [0.79,1.25] | 1.61 [1.29,2.02] | 1.62 [1.29,2.03] | 1.00 [0.83,1.21] | 1.01 [0.84,1.22] |
| | 13-17 | 1.06 [0.77,1.45] | 1.04 [0.74,1.48] | 1.36 [1.09,1.71] | 1.44 [1.13,1.85] | 2.29 [1.84,2.84] | 2.48 [1.93,3.18] | 1.33 [1.11,1.61] | 1.71 [1.39,2.11] |
| | 18-24 | 2.31 [1.75,3.06] | 2.25 [1.70,2.99] | 1.88 [1.50,2.36] | 1.91 [1.52,2.40] | 1.74 [1.34,2.27] | 1.81 [1.39,2.36] | 1.62 [1.33,1.96] | 1.73 [1.43,2.11] |
| | 25-34 | 1.04 [0.79,1.38] | 1.05 [0.79,1.38] | 1.33 [1.10,1.61] | 1.33 [1.09,1.61] | 1.15 [0.91,1.45] | 1.13 [0.89,1.42] | 1.22 [1.03,1.44] | 1.19 [1.01,1.41] |
| | 35-44 | Ref | Ref | Ref | Ref | Ref | Ref | Ref | Ref |
| | 45-54 | 1.01 [0.80,1.28] | 1.03 [0.81,1.31] | 1.14 [0.96,1.36] | 1.13 [0.95,1.35] | 1.05 [0.85,1.28] | 1.07 [0.87,1.31] | 1.02 [0.88,1.18] | 1.08 [0.93,1.25] |
| | 55-64 | 0.69 [0.53,0.88] | 0.69 [0.54,0.90] | 1.08 [0.91,1.28] | 1.09 [0.91,1.29] | 0.78 [0.63,0.96] | 0.83 [0.67,1.03] | 0.89 [0.77,1.03] | 1.02 [0.88,1.18] |
| | 65+ | 0.61 [0.48,0.78] | 0.63 [0.48,0.82] | 0.76 [0.64,0.91] | 0.83 [0.68,1.00] | 0.56 [0.45,0.68] | 0.67 [0.54,0.85] | 0.60 [0.52,0.70] | 0.80 [0.67,0.94] |
| Region | North East | 3.46 [2.52,4.76] | 3.44 [2.49,4.75] | 2.28 [1.79,2.91] | 2.17 [1.69,2.78] | 1.47 [1.09,1.97] | 1.28 [0.95,1.73] | 0.74 [0.57,0.96] | 0.68 [0.52,0.89] |
| | North West | 3.92 [3.09,4.98] | 3.92 [3.07,5.00] | 3.15 [2.67,3.70] | 3.01 [2.55,3.55] | 1.34 [1.10,1.64] | 1.20 [0.98,1.47] | 0.84 [0.72,0.98] | 0.78 [0.66,0.91] |
| | Yorkshire | 2.44 [1.81,3.29] | 2.49 [1.85,3.37] | 2.67 [2.20,3.23] | 2.60 [2.14,3.15] | 1.47 [1.17,1.85] | 1.38 [1.10,1.74] | 0.60 [0.48,0.74] | 0.57 [0.45,0.71] |
| | East Midlands | 1.76 [1.34,2.32] | 1.78 [1.35,2.35] | 1.70 [1.42,2.03] | 1.67 [1.40,2.00] | 1.54 [1.28,1.84] | 1.48 [1.23,1.77] | 0.71 [0.60,0.82] | 0.68 [0.58,0.79] |
| | West Midlands | 1.62 [1.20,2.20] | 1.62 [1.19,2.20] | 1.97 [1.63,2.38] | 1.92 [1.59,2.32] | 1.52 [1.24,1.86] | 1.42 [1.16,1.74] | 0.89 [0.76,1.05] | 0.85 [0.73,1.01] |
| | East of England | 1.19 [0.89,1.60] | 1.22 [0.91,1.63] | 0.95 [0.78,1.17] | 0.94 [0.77,1.16] | 0.76 [0.61,0.95] | 0.75 [0.60,0.92] | 1.05 [0.92,1.20] | 1.03 [0.90,1.17] |
| | London | 1.63 [1.21,2.18] | 1.46 [1.08,1.98] | 1.26 [1.03,1.56] | 1.19 [0.96,1.47] | 1.24 [1.01,1.53] | 1.07 [0.86,1.34] | 1.62 [1.42,1.84] | 1.43 [1.24,1.64] |
| | South East | Ref | Ref | Ref | Ref | Ref | Ref | Ref | Ref |
| | South West | 1.32 [0.95,1.83] | 1.39 [1.00,1.93] | 1.15 [0.92,1.43] | 1.14 [0.91,1.42] | 0.84 [0.66,1.07] | 0.82 [0.64,1.04] | 0.52 [0.42,0.63] | 0.50 [0.41,0.61] |
| Ethnic group | White | Ref | Ref | Ref | Ref | Ref | Ref | Ref | Ref |
| | Asian | 1.81 [1.41,2.31] | 2.02 [1.57,2.62] | 1.10 [0.88,1.37] | 1.24 [0.98,1.55] | 1.67 [1.35,2.07] | 1.63 [1.31,2.03] | 1.79 [1.52,2.10] | 1.45 [1.23,1.72] |
| | Black | 1.51 [0.92,2.49] | 1.65 [0.99,2.75] | 1.16 [0.77,1.76] | 1.27 [0.83,1.94] | 1.20 [0.75,1.91] | 1.05 [0.65,1.68] | 2.40 [1.85,3.12] | 1.67 [1.28,2.19] |
| | Mixed | 0.70 [0.38,1.27] | 0.78 [0.43,1.43] | 0.88 [0.60,1.29] | 0.97 [0.66,1.42] | 0.82 [0.54,1.25] | 0.84 [0.55,1.27] | 1.14 [0.85,1.53] | 0.99 [0.73,1.33] |
| | Other | 1.03 [0.49,2.17] | 1.13 [0.53,2.40] | 1.31 [0.81,2.11] | 1.48 [0.91,2.40] | 1.72 [1.04,2.83] | 1.71 [1.04,2.83] | 1.80 [1.24,2.61] | 1.40 [0.96,2.04] |
| | Unknown | 1.09 [0.61,1.92] | 1.12 [0.63,1.99] | 1.20 [0.83,1.73] | 1.25 [0.87,1.80] | 1.49 [1.00,2.22] | 1.45 [0.97,2.16] | 1.20 [0.89,1.61] | 1.11 [0.82,1.49] |
| Deprivation Index Quintile | 1 - Most Deprived | 1.60 [1.29,1.98] | 1.10 [0.88,1.38] | 1.66 [1.42,1.95] | 1.25 [1.06,1.47] | 1.89 [1.58,2.27] | 1.70 [1.41,2.05] | 1.41 [1.22,1.64] | 1.41 [1.21,1.65] |
| | 2 | 1.00 [0.81,1.25] | 0.86 [0.69,1.07] | 1.20 [1.03,1.39] | 1.08 [0.92,1.25] | 1.41 [1.19,1.68] | 1.34 [1.13,1.60] | 1.47 [1.29,1.66] | 1.39 [1.22,1.58] |
| | 3 | 0.85 [0.69,1.05] | 0.80 [0.65,0.99] | 1.08 [0.94,1.25] | 1.04 [0.90,1.20] | 1.19 [1.01,1.40] | 1.17 [0.99,1.38] | 1.15 [1.01,1.30] | 1.14 [1.00,1.29] |
| | 4 | 0.97 [0.80,1.18] | 0.91 [0.75,1.10] | 1.08 [0.95,1.24] | 1.02 [0.89,1.17] | 1.11 [0.94,1.30] | 1.09 [0.93,1.28] | 1.12 [0.99,1.26] | 1.13 [1.00,1.28] |
| | 5 - Least Deprived | Ref | Ref | Ref | Ref | Ref | Ref | Ref | Ref |
| Work type 1 | 1. Health care workers with direct patient contact | 1.36 [0.98,1.88] | 1.26 [0.91,1.75] | 1.00 [0.79,1.26] | 0.98 [0.77,1.24] | 1.55 [1.22,1.95] | 1.49 [1.17,1.88] | 1.23 [1.01,1.50] | 1.16 [0.95,1.41] |
| | 2. Health care workers with no patient contact | 0.77 [0.38,1.57] | 0.74 [0.36,1.51] | 0.69 [0.43,1.11] | 0.69 [0.43,1.11] | 1.03 [0.64,1.67] | 1.03 [0.64,1.66] | 0.44 [0.26,0.74] | 0.42 [0.24,0.71] |
| | 3. Care home workers with direct contact with clients | 0.85 [0.31,2.29] | 0.78 [0.29,2.10] | 1.59 [0.97,2.60] | 1.53 [0.93,2.50] | 1.64 [0.91,2.93] | 1.51 [0.84,2.71] | 2.15 [1.46,3.17] | 2.02 [1.37,2.98] |
| | 4. Care home workers without contact with clients | 1.26 [0.17,9.03] | 1.31 [0.18,9.47] | 1.49 [0.47,4.70] | 1.55 [0.49,4.90] | 1.30 [0.32,5.25] | 1.25 [0.31,5.06] | 3.60 [1.83,7.11] | 3.52 [1.78,6.95] |
| | 5. Other essential/key workers | Ref | Ref | Ref | Ref | Ref | Ref | Ref | Ref |
| | 6. None of these | 0.93 [0.77,1.12] | 0.97 [0.81,1.17] | 0.80 [0.70,0.90] | 0.84 [0.74,0.96] | 0.80 [0.70,0.93] | 0.81 [0.70,0.94] | 0.75 [0.67,0.83] | 0.71 [0.64,0.78] |
| | 7. Don't know | 1.03 [0.59,1.78] | 0.97 [0.56,1.68] | 1.17 [0.83,1.64] | 1.16 [0.83,1.64] | 1.32 [0.89,1.95] | 1.25 [0.84,1.85] | 0.73 [0.51,1.04] | 0.69 [0.48,0.99] |
| Work type 2 | 1. Delivering to homes - No | Ref | Ref | Ref | Ref | Ref | Ref | Ref | Ref |
| | Delivering to homes - Yes | 0.78 [0.37,1.66] | 0.79 [0.37,1.66] | 0.88 [0.54,1.45] | 0.86 [0.52,1.41] | 1.15 [0.69,1.93] | 1.13 [0.68,1.89] | 1.13 [0.79,1.63] | 1.15 [0.80,1.65] |
| | 2. Food retail, other shop work - No | Ref | Ref | Ref | Ref | Ref | Ref | Ref | Ref |
| | Food retail, other shop work - Yes | 1.20 [0.89,1.61] | 1.15 [0.86,1.56] | 1.33 [1.08,1.64] | 1.28 [1.03,1.57] | 1.26 [0.97,1.64] | 1.20 [0.92,1.56] | 1.28 [1.06,1.53] | 1.30 [1.08,1.56] |
| | 10. Hospitality - No | Ref | Ref | Ref | Ref | Ref | Ref | Ref | Ref |
| | Hospitality - Yes | 1.34 [0.93,1.93] | 1.28 [0.89,1.85] | 1.23 [0.92,1.64] | 1.20 [0.90,1.60] | 1.68 [1.23,2.30] | 1.64 [1.19,2.24] | 1.15 [0.88,1.51] | 1.20 [0.92,1.56] |
| | 11. Personal care - No | Ref | Ref | Ref | Ref | Ref | Ref | Ref | Ref |
| | Personal care - Yes | 0.73 [0.32,1.63] | 0.75 [0.34,1.69] | 1.50 [0.98,2.30] | 1.56 [1.01,2.38] | 1.05 [0.58,1.91] | 1.06 [0.58,1.94] | 0.78 [0.48,1.26] | 0.81 [0.50,1.30] |
| | 3. Policing, prisons, fire & rescue, coastguard - No | Ref | Ref | Ref | Ref | Ref | Ref | Ref | Ref |
| | Policing, prisons, fire & rescue, coastguard - Yes | 0.83 [0.37,1.86] | 0.84 [0.38,1.89] | 1.01 [0.62,1.66] | 0.98 [0.60,1.62] | 1.92 [1.22,3.00] | 2.02 [1.29,3.16] | 1.56 [1.09,2.23] | 1.68 [1.17,2.41] |
| | 4. Public transport - No | Ref | Ref | Ref | Ref | Ref | Ref | Ref | Ref |
| | Public transport - Yes | 1.43 [0.76,2.69] | 1.38 [0.74,2.60] | 0.76 [0.41,1.43] | 0.75 [0.40,1.40] | 1.50 [0.84,2.66] | 1.39 [0.78,2.46] | 2.51 [1.84,3.43] | 2.17 [1.58,2.97] |
| | 5. Education, school, nursery or childcare - No | Ref | Ref | Ref | Ref | Ref | Ref | Ref | Ref |
| | Education, school, nursery or childcare - Yes | 1.16 [0.91,1.49] | 1.17 [0.91,1.50] | 1.23 [1.04,1.45] | 1.22 [1.04,1.44] | 1.12 [0.92,1.37] | 1.14 [0.94,1.40] | 1.16 [1.00,1.35] | 1.20 [1.03,1.39] |
| | 12. Childcare - No | Ref | Ref | Ref | Ref | Ref | Ref | Ref | Ref |
| | Childcare - Yes | NA | NA | NA | NA | NA | NA | NA | NA |
| | 6. Armed forces - No | Ref | Ref | Ref | Ref | Ref | Ref | Ref | Ref |
| | Armed forces - Yes | NA | NA | NA | NA | 0.63 [0.09,4.53] | 0.67 [0.09,4.80] | 1.67 [0.69,4.07] | 1.84 [0.76,4.48] |
| | 7. Another public facing role - No | Ref | Ref | Ref | Ref | Ref | Ref | Ref | Ref |
| | Another public facing role - Yes | 0.98 [0.77,1.26] | 0.97 [0.76,1.25] | 1.19 [1.01,1.40] | 1.18 [1.00,1.38] | 1.17 [0.96,1.43] | 1.16 [0.95,1.42] | 1.18 [1.03,1.36] | 1.21 [1.05,1.39] |

| | | | | | | | | |
|---|---|---|---|---|---|---|---|---|
| 8. Work outside but not in public facing role - No | Ref | Ref | Ref | Ref | Ref | Ref | Ref | Ref |
| Work outside but not in public facing role - Yes | 1.08 [0.89,1.32] | 1.12 [0.92,1.36] | 1.04 [0.91,1.20] | 1.04 [0.91,1.20] | 1.01 [0.85,1.20] | 1.01 [0.85,1.21] | 1.06 [0.94,1.20] | 1.12 [0.99,1.27] |
| 9. Not currently required to work outside my home - | Ref | Ref | Ref | Ref | Ref | Ref | Ref | Ref |
| Not currently required to work outside my home- Yes | 0.84 [0.72,0.98] | 0.83 [0.71,0.98] | 0.74 [0.67,0.83] | 0.76 [0.68,0.85] | 0.74 [0.65,0.84] | 0.74 [0.65,0.84] | 0.71 [0.65,0.78] | 0.67 [0.61,0.74] |

| Round 9 | | Round 10 | | Rounds 5 to 10 | |
|---|---|---|---|---|---|
| Adjusted for age and sex | Mutually adjusted | Adjusted for age and sex | Mutually adjusted | Adjusted for age and sex | Mutually adjusted |
| Ref | Ref | Ref | Ref | Ref | Ref |
| 0.97 [0.83,1.12] | 0.96 [0.82,1.12] | 0.87 [0.67,1.14] | 0.88 [0.67,1.15] | 0.95 [0.91, 1.00] | 0.95 [0.90, 0.99] |
| 1.33 [0.99,1.80] | 1.38 [1.02,1.86] | 1.50 [0.93,2.42] | 1.48 [0.91,2.39] | 1.15 [0.93, 1.43] | 1.16 [0.94, 1.45] |
| 0.99 [0.70,1.42] | 1.12 [0.76,1.65] | 0.87 [0.47,1.63] | 0.79 [0.40,1.57] | 1.30 [0.99, 1.71] | 1.41 [1.04, 1.92] |
| 1.33 [0.91,1.92] | 1.40 [0.96,2.04] | 1.11 [0.59,2.11] | 1.09 [0.57,2.08] | 1.73 [1.49, 2.01] | 1.80 [1.61, 2.01] |
| 1.03 [0.75,1.41] | 1.01 [0.74,1.38] | 0.86 [0.50,1.47] | 0.83 [0.48,1.42] | 1.18 [1.07, 1.30] | 1.16 [1.06, 1.28] |
| Ref | Ref | Ref | Ref | Ref | Ref |
| 1.07 [0.82,1.40] | 1.11 [0.85,1.45] | 0.82 [0.52,1.30] | 0.83 [0.52,1.33] | 1.05 [0.96, 1.14] | 1.08 [0.99, 1.17] |
| 0.95 [0.73,1.24] | 1.04 [0.79,1.36] | 0.74 [0.47,1.17] | 0.77 [0.48,1.22] | 0.87 [0.75, 1.00] | 0.92 [0.80, 1.06] |
| 0.53 [0.40,0.70] | 0.64 [0.46,0.87] | 0.44 [0.28,0.71] | 0.48 [0.29,0.81] | 0.60 [0.53, 0.69] | 0.71 [0.63, 0.81] |
| 2.20 [1.54,3.14] | 1.89 [1.32,2.72] | 5.75 [2.93,11.28] | 5.28 [2.67,10.46] | 2.11 [1.22, 3.65] | 1.95 [1.11, 3.41] |
| 1.81 [1.40,2.34] | 1.60 [1.23,2.09] | 4.51 [2.59,7.85] | 4.18 [2.38,7.34] | 2.16 [1.28, 3.65] | 2.01 [1.16, 3.48] |
| 1.17 [0.82,1.67] | 1.09 [0.76,1.55] | 5.16 [2.85,9.33] | 5.00 [2.75,9.08] | 1.77 [0.98, 3.17] | 1.70 [0.94, 3.10] |
| 1.51 [1.16,1.97] | 1.44 [1.10,1.88] | 3.33 [1.88,5.89] | 3.27 [1.85,5.81] | 1.53 [1.06, 2.23] | 1.50 [1.02, 2.19] |
| 1.20 [0.88,1.64] | 1.11 [0.81,1.52] | 3.53 [1.93,6.44] | 3.41 [1.86,6.23] | 1.55 [1.12, 2.16] | 1.49 [1.06, 2.09] |
| 1.12 [0.85,1.48] | 1.09 [0.83,1.45] | 2.35 [1.29,4.27] | 2.31 [1.27,4.19] | 1.06 [0.87, 1.30] | 1.05 [0.86, 1.30] |
| 1.73 [1.32,2.28] | 1.52 [1.14,2.02] | 2.52 [1.35,4.70] | 2.16 [1.14,4.10] | 1.51 [1.31, 1.73] | 1.34 [1.17, 1.52] |
| Ref | Ref | Ref | Ref | Ref | Ref |
| 0.66 [0.46,0.97] | 0.65 [0.44,0.94] | 1.90 [0.95,3.76] | 1.89 [0.95,3.77] | 0.93 [0.65, 1.32] | 0.92 [0.64, 1.34] |
| Ref | Ref | Ref | Ref | Ref | Ref |
| 1.81 [1.36,2.42] | 1.65 [1.22,2.21] | 1.31 [0.76,2.27] | 1.36 [0.77,2.37] | 1.58 [1.32, 1.89] | 1.55 [1.34, 1.78] |
| 1.48 [0.87,2.52] | 1.15 [0.67,1.98] | 1.13 [0.42,3.06] | 1.06 [0.39,2.93] | 1.52 [1.13, 2.03] | 1.39 [1.14, 1.69] |
| 0.58 [0.29,1.16] | 0.55 [0.27,1.12] | 1.41 [0.66,3.03] | 1.56 [0.72,3.36] | 0.92 [0.75, 1.13] | 0.92 [0.77, 1.11] |
| 0.97 [0.40,2.36] | 0.86 [0.36,2.10] | 1.52 [0.49,4.78] | 1.58 [0.50,5.02] | 1.50 [1.20, 1.89] | 1.41 [1.12, 1.77] |
| 1.34 [0.79,2.28] | 1.27 [0.75,2.17] | 1.44 [0.59,3.52] | 1.43 [0.59,3.49] | 1.26 [1.06, 1.50] | 1.23 [1.03, 1.46] |
| 1.95 [1.52,2.49] | 1.67 [1.29,2.15] | 2.00 [1.31,3.06] | 1.44 [0.93,2.23] | 1.69 [1.51, 1.89] | 1.41 [1.22, 1.62] |
| 1.62 [1.29,2.03] | 1.48 [1.17,1.86] | 1.57 [1.05,2.33] | 1.35 [0.91,2.02] | 1.34 [1.17, 1.54] | 1.22 [1.04, 1.44] |
| 1.01 [0.79,1.27] | 0.98 [0.77,1.24] | 1.10 [0.74,1.64] | 1.01 [0.67,1.50] | 1.07 [0.98, 1.18] | 1.03 [0.92, 1.16] |
| 1.12 [0.90,1.40] | 1.09 [0.88,1.37] | 1.05 [0.71,1.56] | 0.96 [0.65,1.43] | 1.09 [1.02, 1.16] | 1.06 [0.98, 1.13] |
| Ref | Ref | Ref | Ref | Ref | Ref |
| 1.26 [0.90,1.78] | 1.23 [0.87,1.73] | 1.32 [0.73,2.38] | 1.30 [0.72,2.35] | 1.24 [1.09, 1.40] | 1.18 [1.04, 1.33] |
| 0.88 [0.45,1.72] | 0.89 [0.45,1.74] | 0.61 [0.15,2.51] | 0.62 [0.15,2.56] | 0.78 [0.61, 1.00] | 0.77 [0.60, 0.99] |
| | | | | | |
| 0.87 [0.32,2.36] | 0.81 [0.30,2.18] | 1.33 [0.32,5.48] | 1.24 [0.30,5.15] | 1.45 [1.09, 1.93] | 1.35 [1.01, 1.81] |
| 3.15 [1.00,9.97] | 3.04 [0.96,9.64] | NA | NA | 2.51 [1.62, 3.89] | 2.44 [1.57, 3.79] |
| Ref | Ref | Ref | Ref | Ref | Ref |
| 0.83 [0.69,1.00] | 0.83 [0.69,1.00] | 1.00 [0.71,1.41] | 1.07 [0.76,1.51] | 0.83 [0.77, 0.89] | 0.84 [0.77, 0.92] |
| 0.94 [0.51,1.73] | 0.89 [0.48,1.64] | 1.79 [0.76,4.19] | 1.73 [0.73,4.06] | 1.20 [0.92, 1.57] | 1.15 [0.88, 1.51] |
| Ref | Ref | Ref | Ref | Ref | Ref |
| 0.73 [0.33,1.64] | 0.72 [0.32,1.61] | 1.94 [0.79,4.73] | 1.84 [0.75,4.50] | 1.05 [0.84, 1.31] | 1.04 [0.83, 1.30] |
| Ref | Ref | Ref | Ref | Ref | Ref |
| 1.17 [0.80,1.71] | 1.13 [0.77,1.65] | 1.18 [0.62,2.25] | 1.08 [0.57,2.05] | 1.27 [1.14, 1.41] | 1.24 [1.11, 1.38] |
| Ref | Ref | Ref | Ref | Ref | Ref |
| 0.97 [0.50,1.87] | 0.92 [0.47,1.78] | 0.26 [0.04,1.83] | 0.24 [0.03,1.71] | 1.28 [1.09, 1.50] | 1.27 [1.09, 1.47] |
| Ref | Ref | Ref | Ref | Ref | Ref |
| 0.45 [0.11,1.81] | 0.47 [0.12,1.87] | 0.58 [0.08,4.17] | 0.57 [0.08,4.09] | 0.96 [0.68, 1.36] | 0.99 [0.69, 1.40] |
| Ref | Ref | Ref | Ref | Ref | Ref |
| 1.42 [0.73,2.75] | 1.49 [0.77,2.90] | 1.67 [0.53,5.27] | 1.66 [0.53,5.22] | 1.41 [1.1, 1.81] | 1.44 [1.09, 1.92] |
| Ref | Ref | Ref | Ref | Ref | Ref |
| 2.42 [1.36,4.31] | 2.14 [1.20,3.83] | 1.64 [0.52,5.17] | 1.54 [0.49,4.86] | 1.65 [1.13, 2.43] | 1.53 [1.08, 2.17] |
| Ref | Ref | Ref | Ref | Ref | Ref |
| 1.40 [1.04,1.87] | 1.43 [1.07,1.91] | 1.09 [0.66,1.81] | 1.10 [0.66,1.83] | 1.19 [1.09, 1.29] | 1.21 [1.11, 1.31] |
| Ref | Ref | Ref | Ref | Ref | Ref |
| 0.58 [0.14,2.32] | 0.57 [0.14,2.29] | 1.60 [0.39,6.46] | 1.69 [0.42,6.83] | 0.96 [0.35, 2.59] | 0.98 [0.34, 2.83] |
| Ref | Ref | Ref | Ref | Ref | Ref |
| 2.11 [0.52,8.51] | 2.24 [0.55,9.07] | NA | NA | 1.57 [0.78, 3.16] | 1.70 [0.84, 3.43] |
| Ref | Ref | Ref | Ref | Ref | Ref |
| 1.23 [0.94,1.60] | 1.22 [0.93,1.59] | 0.98 [0.60,1.62] | 0.95 [0.57,1.57] | 1.16 [1.07, 1.26] | 1.16 [1.07, 1.26] |

| | | | | | |
|---|---|---|---|---|---|
| Ref | Ref | Ref | Ref | Ref | Ref |
| 1.45 [1.17,1.80] | 1.48 [1.19,1.83] | 1.34 [0.91,1.95] | 1.30 [0.89,1.90] | 1.11 [1.01, 1.23] | 1.13 [1.02, 1.26] |
| Ref | Ref | Ref | Ref | Ref | Ref |
| 0.64 [0.54,0.76] | 0.64 [0.54,0.76] | 0.82 [0.61,1.10] | 0.86 [0.64,1.16] | 0.73 [0.70, 0.78] | 0.73 [0.68, 0.79] |
