# Supplementary material for: Health inequalities in risk of SARS-CoV-2 infection in England’s second wave: population-based cross-sectional analysis from the REACT-1 study
Source: BMJ Public Health. 2026 Jan 5;4(1):e002596. doi: 10.1136/bmjph-2025-002596 (PMC13663695; doi:10.1136/bmjph-2025-002596)
Supplement: Supplementary file 9 [file bmjph-4-1-s009.pdf]

Supplementary Table 8. Odds ratios for rounds 9 and 10, and an average weighted inversely to variance for linked vaccination data.

Variable	Category	Round 9		Round 10		Rounds 9 to 10	
		Mutually adjusted (linked data)**	Mutually adjusted (including vaccination status)	Mutually adjusted (linked data)**	Mutually adjusted (including vaccination status)	Mutually adjusted (linked data)	Mutually adjusted (including vaccination status)
Sex	Male	Ref	Ref	Ref	Ref	Ref	Ref
	Female	1.09 [0.91,1.32]	1.12 [0.93,1.35]	0.86 [0.62,1.19]	0.86 [0.62,1.19]	1.01 [0.8, 1.26]	1.02 [0.79, 1.31]
Age Group	5-12	1.19 [0.82,1.71]	0.94 [0.64,1.37]	1.08 [0.60,1.96]	1.07 [0.58,1.96]	1.16 [0.85, 1.58]	0.97 [0.71, 1.34]
	13-17	1.04 [0.64,1.69]	0.87 [0.53,1.41]	0.78 [0.34,1.79]	0.76 [0.33,1.77]	0.97 [0.64, 1.47]	0.84 [0.55, 1.28]
	18-24	1.17 [0.73,1.88]	1.08 [0.67,1.73]	0.87 [0.39,1.93]	0.86 [0.38,1.91]	1.08 [0.72, 1.63]	1.02 [0.68, 1.53]
	25-34	1.07 [0.74,1.55]	1.03 [0.71,1.49]	0.67 [0.34,1.34]	0.67 [0.34,1.33]	0.93 [0.61, 1.42]	0.92 [0.63, 1.33]
	35-44	Ref	Ref	Ref	Ref	Ref	Ref
	45-54	1.10 [0.80,1.50]	1.24 [0.91,1.70]	0.73 [0.42,1.25]	0.74 [0.43,1.28]	0.95 [0.65, 1.4]	1.01 [0.62, 1.66]
	55-64	1.14 [0.82,1.57]	1.50 [1.06,2.12]	0.74 [0.43,1.29]	0.79 [0.43,1.46]	0.98 [0.65, 1.46]	1.15 [0.62, 2.13]
	65+	0.69 [0.46,1.06]	1.27 [0.79,2.05]	0.39 [0.19,0.79]	0.38 [0.17,0.86]	0.56 [0.33, 0.96]	0.73 [0.23, 2.37]
Region	North East	1.91 [1.22,2.98]	1.88 [1.20,2.94]	4.49 [1.90,10.60]	4.48 [1.89,10.58]	2.69 [1.18, 6.13]	2.67 [1.16, 6.18]
	North West	1.80 [1.31,2.48]	1.82 [1.32,2.51]	4.45 [2.28,8.66]	4.43 [2.27,8.62]	2.69 [1.12, 6.5]	2.7 [1.14, 6.41]
	Yorkshire and The Humber	1.17 [0.76,1.81]	1.17 [0.76,1.80]	5.27 [2.61,10.66]	5.26 [2.60,10.64]	2.42 [0.56, 10.54]	2.41 [0.55, 10.53]
	East Midlands	1.68 [1.23,2.31]	1.69 [1.23,2.32]	3.07 [1.54,6.11]	3.08 [1.55,6.12]	2.1 [1.19, 3.71]	2.1 [1.19, 3.71]
	West Midlands	1.20 [0.82,1.75]	1.22 [0.84,1.79]	2.88 [1.37,6.09]	2.89 [1.37,6.11]	1.75 [0.75, 4.1]	1.77 [0.77, 4.07]
	East of England	1.19 [0.85,1.67]	1.20 [0.86,1.68]	2.20 [1.07,4.49]	2.19 [1.07,4.48]	1.49 [0.84, 2.64]	1.49 [0.85, 2.62]
	London	1.49 [1.04,2.13]	1.52 [1.06,2.18]	2.77 [1.31,5.88]	2.76 [1.30,5.85]	1.86 [1.04, 3.33]	1.86 [1.07, 3.25]
	South East	Ref	Ref	Ref	Ref	Ref	Ref
	South West	0.82 [0.54,1.26]	0.82 [0.54,1.26]	2.10 [0.95,4.62]	2.10 [0.96,4.62]	1.24 [0.5, 3.07]	1.24 [0.5, 3.08]
Key Worker Status	HCW/CHW*	1.52 [1.08,2.13]	2.39 [1.62,3.53]	1.33 [0.73,2.45]	1.29 [0.66,2.51]	1.47 [1.09, 1.98]	1.87 [1.03, 3.38]
	Other essential/key workers	1.18 [0.92,1.50]	1.23 [0.96,1.57]	1.08 [0.69,1.70]	1.08 [0.69,1.69]	1.15 [0.93, 1.43]	1.19 [0.96, 1.48]
	Other worker	Ref	Ref	Ref	Ref	Ref	Ref
	Not FT, PT, SE*	0.94 [0.72,1.22]	1.01 [0.77,1.32]	1.10 [0.69,1.75]	1.11 [0.69,1.76]	0.98 [0.78, 1.23]	1.03 [0.82, 1.3]
	Unknown	1.30 [0.64,2.67]	1.33 [0.65,2.73]	3.34 [1.42,7.88]	3.34 [1.42,7.88]	2.02 [0.81, 5.08]	2.05 [0.83, 5.02]
Ethnicity	White	Ref	Ref	Ref	Ref	Ref	Ref
	Asian	1.89 [1.31,2.72]	1.91 [1.32,2.76]	0.80 [0.32,2.00]	0.80 [0.32,2.00]	1.37 [0.61, 3.08]	1.38 [0.6, 3.13]
	Black	0.62 [0.23,1.68]	0.60 [0.22,1.62]	1.61 [0.50,5.20]	1.62 [0.50,5.24]	0.95 [0.38, 2.39]	0.93 [0.35, 2.48]
	Mixed	0.42 [0.16,1.13]	0.42 [0.15,1.12]	1.33 [0.48,3.67]	1.33 [0.49,3.67]	0.75 [0.24, 2.31]	0.74 [0.24, 2.32]
	Other	0.91 [0.29,2.85]	0.90 [0.29,2.83]	0.87 [0.12,6.32]	0.87 [0.12,6.31]	0.9 [0.33, 2.42]	0.89 [0.33, 2.41]
	Unknown	1.71 [0.91,3.22]	1.71 [0.91,3.21]	1.57 [0.50,4.96]	1.58 [0.50,4.97]	1.68 [0.96, 2.92]	1.68 [0.96, 2.92]
Household Size	1-2 People	Ref	Ref	Ref	Ref	Ref	Ref
	3-5 People	1.55 [1.24,1.94]	1.52 [1.22,1.91]	1.31 [0.88,1.96]	1.31 [0.88,1.96]	1.49 [1.22, 1.81]	1.47 [1.21, 1.79]
	6+ People	1.78 [1.09,2.91]	1.76 [1.07,2.88]	2.00 [0.90,4.44]	2.01 [0.90,4.45]	1.84 [1.21, 2.8]	1.82 [1.2, 2.77]
Deprivation Index Quintile	1 - Most Deprived	1.95 [1.45,2.63]	1.95 [1.44,2.63]	0.98 [0.56,1.74]	0.98 [0.56,1.75]	1.45 [0.75, 2.82]	1.45 [0.75, 2.81]
	2	1.55 [1.17,2.04]	1.54 [1.17,2.04]	1.00 [0.61,1.65]	1.00 [0.61,1.65]	1.31 [0.87, 1.98]	1.31 [0.87, 1.98]
	3	0.85 [0.63,1.15]	0.85 [0.63,1.14]	0.93 [0.59,1.48]	0.93 [0.59,1.48]	0.87 [0.68, 1.12]	0.87 [0.68, 1.12]
	4	1.07 [0.82,1.39]	1.07 [0.82,1.40]	0.89 [0.57,1.40]	0.89 [0.57,1.40]	1.02 [0.81, 1.28]	1.02 [0.81, 1.28]
	5 - Least Deprived	Ref	Ref	Ref	Ref	Ref	Ref
Vaccination status	Unvaccinated	Ref	Ref	Ref	Ref	Ref	Ref
	Vaccinated - 1 dose	NA	0.57 [0.44,0.73]	NA	0.82 [0.47,1.42]	NA	0.63 [0.45, 0.86]
	Vaccinated - 2 doses	NA	0.38 [0.26,0.55]	NA	1.01 [0.59,1.74]	NA	0.61 [0.23, 1.59]

* HCW/CHW: health care worker/care home worker; Not FT, PT SE: not self-employed, full-time, part-time, self-employed.

**The sample size for linked vaccination data is smaller than REACT data used in previous regression analysis. The sample size for round 9 is 114,747, and for round 10 is 98,983.
